# *Trem2* H157Y increases soluble TREM2 production and reduces amyloid pathology

**DOI:** 10.1186/s13024-023-00599-3

**Published:** 2023-01-31

**Authors:** Wenhui Qiao, Yixing Chen, Jun Zhong, Benjamin J. Madden, Cristine M. Charlesworth, Yuka A. Martens, Chia-Chen Liu, Joshua Knight, Tadafumi C. Ikezu, Aishe Kurti, Yiyang Zhu, Axel Meneses, Cassandra L. Rosenberg, Lindsey A. Kuchenbecker, Lucy K. Vanmaele, Fuyao Li, Kai Chen, Francis Shue, Maxwell V. Dacquel, John Fryer, Akhilesh Pandey, Na Zhao, Guojun Bu

**Affiliations:** 1 Department of Neuroscience, Mayo Clinic, Jacksonville, FL 32224 USA; 2 Department of Laboratory Medicine and Pathology, Mayo Clinic, Rochester, MN 55905 USA; 3 Medical Genome Facility, Proteomics Core, Mayo Clinic, Rochester, MN USA; 4 Department of Neuroscience, Mayo Clinic, Scottsdale, AZ 85259 USA; 5 Center for Individualized Medicine, Mayo Clinic, Rochester, MN USA; 6 Manipal Academy of Higher Education (MAHE), Manipal, Karnataka 576104 India; 7 SciNeuro Pharmaceuticals, Rockville, MD 20805 USA

**Keywords:** *Trem2* H157Y mutation, Soluble TREM2, Microglia, Synaptic plasticity, Amyloid pathology, Aβ clearance

## Abstract

**Background:**

The rare p.H157Y variant of *TREM2* (Triggering Receptor Expressed on Myeloid Cells 2) was found to increase Alzheimer’s disease (AD) risk. This mutation is located at the cleavage site of TREM2 extracellular domain. Ectopic expression of TREM2-H157Y in HEK293 cells resulted in increased TREM2 shedding. However, the physiological outcomes of the *TREM2* H157Y mutation remain unknown in the absence and presence of AD related pathologies.

**Methods:**

We generated a novel *Trem2* H157Y knock-in mouse model through CRISPR/Cas9 technology and investigated the effects of *Trem2* H157Y on TREM2 proteolytic processing, synaptic function, and AD-related amyloid pathologies by conducting biochemical assays, targeted mass spectrometry analysis of TREM2, hippocampal electrophysiology, immunofluorescent staining, in vivo micro-dialysis, and cortical bulk RNA sequencing.

**Results:**

Consistent with previous in vitro findings, *Trem2* H157Y increases TREM2 shedding with elevated soluble TREM2 levels in the brain and serum. Moreover, *Trem2* H157Y enhances synaptic plasticity without affecting microglial density and morphology, or TREM2 signaling. In the presence of amyloid pathology, *Trem2* H157Y accelerates amyloid-β (Aβ) clearance and reduces amyloid burden, dystrophic neurites, and gliosis in two independent founder lines. Targeted mass spectrometry analysis of TREM2 revealed higher ratios of soluble to full-length TREM2-H157Y compared to wild-type TREM2, indicating that the H157Y mutation promotes TREM2 shedding in the presence of Aβ. TREM2 signaling was further found reduced in *Trem2 *H157Y homozygous mice. Transcriptomic profiling revealed that *Trem2* H157Y downregulates neuroinflammation-related genes and an immune module correlated with the amyloid pathology.

**Conclusion:**

Taken together, our findings suggest beneficial effects of the *Trem2* H157Y mutation in synaptic function and in mitigating amyloid pathology. Considering the genetic association of *TREM2* p.H157Y with AD risk, we speculate *TREM2* H157Y in humans might increase AD risk through an amyloid-independent pathway, such as its effects on tauopathy and neurodegeneration which merit further investigation.

**Supplementary Information:**

The online version contains supplementary material available at 10.1186/s13024-023-00599-3.

## Background

Alzheimer’s disease (AD) is a chronic neurodegenerative disease characterized by the pathological deposition of extracellular amyloid plaques and intraneuronal hyperphosphorylated tau tangles, as well as a prominent microglia activation responding to neuropathology and neurodegeneration [1–3]. Multiple microglial gene variants are found to be associated with AD risk [4]. Among them, the p.H157Y variant of Triggering Receptor Expressed on Myeloid Cells 2 (*TREM2*) was identified from a relatively small number of carriers and conferred an increased AD risk with an odds ratio (OR) of 11.01 (minor allele frequency (MAF), 0.4%) in a Han Chinese cohort [5], whereas in a Caucasian cohort used in the Alzheimer’s Disease Sequencing Project, the OR was 4.7 (MAF, 0.06%) [6]. However, how this rare *TREM2* variant contributes to AD risk is not clear.

TREM2 is an immunoreceptor exclusively expressed in microglia in the central nervous system and in myeloid cells (e.g., macrophage) in the periphery [7]. It consists of an Ig-like V type domain, a stalk region, a transmembrane domain, and a short cytoplasmic tail [8]. Most AD-risk variants of *TREM2* (e.g., p.R47H, p.R62H) are located in exon2 which encodes the Ig-like domain [9–11]. These pathogenic mutations majoritively lead to inefficient binding of ligands such as amyloid-β (Aβ) oligomers [12–14], fibrillar Aβ-associated anionic lipids [15], LDL [6, 16], HDL [6], and apolipoproteins [16, 17]. These impairments are associated with microglial dysfunction in phagocytosis in vitro [16, 18, 19] and amyloid plaque engulfment in vivo [20, 21]. In contrast, the p.H157Y variant is located in exon3 which encodes the stalk region. Intriguingly, the H157-S158 site was identified as the ADAM10/17 cleavage site where soluble TREM2 (sTREM2) is produced [22–24]. Ectopic expression of TREM2-H157Y in the HEK293 cells was found to increase sTREM2 in the conditioned medium and reduce membrane-associated mature full-length TREM2 [23, 24]. The increased TREM2 shedding might be related to impaired phagocytosis of pHrodo-E.Coli in HEK293 cells [23] and decreased TREM2 signaling activation in response to phosphatidylserine in 2B4 T cells [6]. Despite these in vitro observations, the in vivo consequences of the *TREM2* H157Y mutation remain unknown.

To address this, we generated a novel *Trem2* H157Y knock-in mouse model through CRISPR/Cas9 technology, and investigated effects of this mutation on TREM2 proteolytic processing, synaptic function, and AD-related amyloid pathology. We found *Trem2* H157Y increased TREM2 shedding, and enhanced synaptic plasticity. In the presence of amyloid pathology, *Trem2* H157Y accelerated Aβ clearance, and reduced amyloid burden, toxic Aβ oligomer, dystrophic neurites, and gliosis at the late stage of amyloid pathology. *Trem2* H157Y was also found to increase TREM2 shedding and reduce TREM2 signaling in the presence of Aβ. In line with these observations, transcriptomic analysis revealed downregulated immune responses which was correlated with reduced amyloid pathology in the *Trem2* H157Y homozygous knock-in mice. Overall, our results imply that *Trem2* H157Y might be beneficial to brain function and in reducing amyloid pathology and related toxicity.

## Methods

### Generation, genotyping, and off-target analysis of *Trem2* H157Y knock-in mice

*Trem2* H157Y knock-in mice were generated via CRISPR/Cas9 by the Hope Center Transgenic Vectors Core of the Washington University [25]. CRISPR gRNAs for in vitro testing were identified using CRISPOR (http://crispor.tefor.net/) and synthesized as gBlocks (Integrated DNA Technologies, IDT) with the sequence 5’GGAGGTGCTGTgTTCCACTT3’. In vitro target specific gRNA cleavage activity was validated by transfecting N2A cells with PCR amplified gRNA gblock and Cas9 plasmid DNA (px330, addgene) using ROCHE Xtremegene HP. Cell pools were harvested 48 h later for genomic DNA prep, followed by sanger sequencing of PCR products spanning the gRNA/Cas9 cleavage site, and TIDE analysis (https://tide.nki.nl/) of sequence trace files. CRISPR sgRNA (IDT, 20 ng/ul) and Cas9 (IDT, 50 ng/ul) proteins were complexed to generate the ribonucleoprotein (RNP) for injection along with a 200 nucleotide ssODN donor DNA (synthesized by IDT, 20 ng/ul), 5’tatatcttgtcctttgctgatctgtttgccctgggacctccatcc gcagtcactgccagggggtctaagaagggaccactactgtacCTGGAGGTGCTGTaTTCCACTTGGGCACCCTCGAAACTCGATGACTCCTCGGGGACCCAGAGATCTCCAGCATCTTGGTCATCTAGAGGGTctgtaatagacaaaccatgagg3’. All animal work were approved by institutional IACUC protocols. B6/CBA F1 mice at 3–4 weeks of age (The Jackson Laboratory, Bar Harbor ME, USA) were superovulated by intraperitoneal injection of 5 IU pregnant mare serum gonadotropin, followed 48 h later by intraperitoneal injection of 5 IU human chorionic gonadotropin (PMS from SIGMA, HGC from Millipore USA). Mouse zygotes were obtained by breeding B6/CBA stud males with superovulated B6/CBA females at a 1:1 ratio. One-cell fertilized embryos were injected into the pronucleus and cytoplasm of each zygote. Microinjections and mouse transgenesis experiments were performed as described previously [26, 27]. Founder genotyping was through deep sequencing (MiSeq, Ilumina). Mosaic founders were crossed to WT to generate heterozygous F1 offspring, which were also deep sequenced to confirm correctly targeted alleles. In addition, we performed off-target analysis with two heterozygous F1 mice from each of the two founders (1 and 2) using the online tool CRISPOR (http://crispor.tefor.net/) [28]. Three putative sites with top CFD scores above 0.3 were identified and examined by Sanger sequencing (GENEWIZ) of PCR amplification products using extracted genomic DNA. No off targets were identified in mice from both founders.

The genotype of *Trem2* in all the mice used for experiments was characterized by quantitative PCR (qPCR) with Custom TaqMan SNP Genotyping assays (Thermo Fisher). All the mice were housed in a temperature-controlled environment with a 12-h light–dark cycle and free access to food and water. All animal procedures were approved by the Mayo Clinic Institutional Animal Care and Use Committee (IACUC) and in accordance with the National Institutes of Health Guidelines for the Care and Use of Laboratory Animals.

### Introduction of *Trem2* H157Y mutation to 5xFAD amyloid mouse model

*Trem2* H157Y homozygous mice (*Trem2*^*H157Y/H157Y*^) were crossed with 5xFAD mice (The Jackson Laboratory, stock # 34848) to obtain the *5xFAD*; *Trem2*^*H157Y/+*^ offspring. *5xFAD*; *Trem2*^*H157Y/+*^ mice were used to setup breeding cages to establish the littermate cohorts with three genotypes including *5xFAD*; *Trem2*^*+/+*^, *5xFAD*; *Trem2*^*H157Y/+*^, and *5xFAD*; *Trem2*^*H157Y/ H157Y*^. The genotype of 5xFAD mice was characterized through probe-based qPCR with the protocol provided by the Jackson Laboratory. All the 5xFAD mice used as breeders or in our experimental cohorts were hemizygous.

### Hippocampal LTP recordings and analyses

Electrophysiological recordings were performed with littermates of *Trem2* H157Y homozygous mice and WT mice at 6 months of age as previously described [29] with minor modifications. Each mouse was acutely decapitated and the brain was dissected out to conduct transverse slicing in ice-cold cutting solution containing 110 mM sucrose, 60 mM NaCl, 3 mM KCl, 1.25 mM NaH_2_PO_4_, 28 mM NaHCO_3_, 0.6 mM sodium ascorbate, 5 mM glucose, 7 mM MgCl_2_ and 0.5 mM CaCl_2_. Field excitatory post-synaptic potentials (fEPSPs) were obtained from area CA1 stratum radiatum with the use of a glass microelectrode (4-6 mΩ) filled with artificial cerebrospinal fluid (aCSF) containing 125 mM NaCl, 2.5 mM KCl, 1.25 mM NaH_2_PO_4_, 25 mM NaHCO_3_, 25 mM glucose, 1 mM MgCl_2_ and 2 mM CaCl_2_. fEPSPs were evoked through stimulation of the Schaffer collaterals using a 0.1 ms biphasic pulse delivered every 20 s. After a consistent response to a voltage stimulus was established, to obtain the profile for input–output curve (I/O curve), the voltage was increased from 0 mV with a step of 0.5-1 mV for 30 sweeps and the inter-sweep interval is 5 s. For each individual slice, all other stimulation paradigms were induced at the same stimulus voltage which produces the 50–60% of the maximum fEPSP amplitude. Paired-pulse facilitation (PPF) was induced with paired-pulses given with an initial delay of 20 ms and the inter-pulse interval incrementally increased 20 ms until a final delay of 400 ms was reached. The fEPSP baseline responses were then recorded for 20 min. The tetanus used to evoke LTP was a theta-burst stimulation (TBS) protocol consisting of five trains of four pulse bursts at 200 Hz separated by 200 ms, repeated six times with an inter-train interval of 10 s. Following TBS, fEPSPs were recorded for 60 min.

All analyses were performed by customized programming in MATLAB (2021b). The fEPSP slope was calculated within the first 1 ms of the descending domain. I/O curve was presented as the fEPSP slope versus fiber volley amplitude responding to increasing stimulus intensities. PPF strength was examined by the ratio of the second fEPSP slope to the first fEPSP slope in each stimulation pair. Long term potentiation profile was assessed in each minute before and after TBS as the mean fEPSP slope normalized to the mean fEPSP slope of baseline recordings.

### Primary microglia culture

Cortical cells from pups (p1-p3) were isolated, filtered with 100 μm cell strainers (Falcon, 352,360), and plated in T75 flasks (Genesee, 25–209) with high-glucose DMEM medium (Gibco, 11965084) containing 10% Fetal Bovine Serum (FBS). Medium was changed to medium containing 25 ng/mL recombinant mouse GM-CSF (Gemini Bio, 300-308P) the next day. Tails from each pup were kept for genotyping. Five days after cell plating, medium in each flask was replaced with fresh GM-CSF-containing medium. On day 9 or 10, microglia were collected by shaking the flasks at 200–220 rpm at room temperature (RT) for ~ 20 min, resuspended in non-GM-CSF containing medium, and plated into 6-well plates. After 24 h, medium from each well was collected as conditioned medium. Cells were lysed with RIPA buffer (Millipore, 20-188) supplemented with protease inhibitor (cOmplete, Roche) and phosphatase inhibitor (PhosSTOP, Roche) followed by mild agitation at 4 °C for 30 min and centrifugation at 20,000 g at 4 °C for 30 min. Supernatant was collected as RIPA lysate.

### Microglia isolation from adult mice

Microglia were isolated from adult mouse brains as previously described [30]. In brief, mice were transcardinally perfused with 0.01 M PBS followed by cortex dissection from both hemispheres. The cortices were dissociated with papain-based enzyme mix (Miltenyi, 130–092-628) using gentleMACS™ Dissociator (Miltenyi, 130-093-235) followed by debris removal through filtering with the 70-µm cell strainer. Myelin was then removed through magnetic sorting after the incubation with myelin removal beads (Miltenyi, 130-096-731). The obtained single cell suspensions were incubated with CD11B^-^ conjugated beads (Miltenyi, 130-049-601) followed by magnetic sorting for CD11B^+^ microglia. Around 300 K microglia were captured per sample and lysed with RIPA buffer supplemented with the protease and phosphatase inhibitors followed by mild agitation at 4 °C for 30 min and centrifugation at 20,000 g at 4 °C for 30 min. Supernatant was collected as RIPA lysate and subject to Western blotting.

### Tissue preparation for immunofluorescence staining, biochemical assays

Blood samples were collected from mice vena cava after isoflurane induced deep anesthesia, stored at 4 °C overnight and subsequently centrifuged at 1000 g for 10 min to collect the supernatant as serum. After blood collection, mice were transcardinally perfused with 0.01 M PBS and the brains were dissected out. Half of the brain was fixed in 4% paraformaldehyde (PFA, Fisher Scientific) for 24 h, dehydrated with 30% sucrose (Sigma) for 48 h, embedded in O.C.T. compound (SAKURA) and snap-frozen in liquid nitrogen before cryostat sectioning. The other hemisphere was dissected into cortex, hippocampus, midbrain, and cerebellum which were snap-frozen in liquid nitrogen and stored at -80 °C. The cortices were then pulverized (CP02 cryoPREP Automated Dry Pulverizer) and divided into two aliquots: 20–30 mg for RNA extraction and 55–65 mg for protein extraction.

Cortical proteins were extracted sequentially with different lysis buffers. Cortical powder was homogenized in Tris-buffered saline (TBS, Fisher Bioreagents, BP2471-500, 600 µl) supplemented with protease inhibitor (cOmplete, Roche) and phosphatase inhibitor (PhosSTOP, Roche) and subjected to ultracentrifugation at 100,000 g at 4 °C for 1 h. The supernatant was collected as TBS lysate with a protein concentration ~ 3 µg/µl. The pellets were then resuspended in TBSX (TBS plus 1% Triton-X100, 600 µl) supplemented with protease inhibitor and phosphatase inhibitor, homogenized, and mild agitated at 4 °C for 30 min followed by ultracentrifugation at 100,000 g at 4 °C for 1 h. Supernatant was collected as TBSX lysate with a protein concentration ~ 3.5 µg/µl for non-amyloid bearing mice and ~ 5 µg/µl for amyloid bearing mice. For amyloid bearing mice, the pellet was further resuspended in 5 M guanidine hydrochloride (GND, Sigma, 600 µl) followed by sonication and centrifuged at 100,000 g for 1 h at 4 °C. The supernatant was collected as GND lysate with a protein concentration ~ 1.6 µg/µl. Total protein concentration in each lysate was measured (Pierce™ BCA Protein Assay Kit, Cat# 23225) before transferring to 96-well storage plates or 1.5 ml tubes and stored at -80 °C until further analysis.

### Immunofluorescence staining, X34 staining and quantification

The embedded hemispheres were coronally sectioned at a 40 µm thickness. Referencing the mouse brain atlas (Paxinos & Franklin, 2013), sections located from AP -1.7 mm to AP -2.06 mm were selected for the following procedures. First, brain slices were blocked in blocking buffer (5% goat serum plus 0.25% Triton in PBS) for 1 h at RT, then incubated overnight in primary antibody solution at 4 °C. Slices were then incubated in the Alexa Fluor-conjugated secondary antibodies solution (1:1000, Invitrogen) at RT for 2 h. We used the following antibodies against IBA1 (Wako, 019-19741, 1:1000), Aβ (MOAB2, Abcam, ab126649, 1:1000), CD68 (Bio-Rad, MCA1957,1:500), LAMP1 (Abcam, ab25245, 1:500), APP (Millipore Sigma, MAB348, 1:300), and GFAP (Millipore Sigma, MAB360, 1:500). To detect TREM2, 5% BSA and 0.25% Triton X-100 in PBS was used for blocking and preparation of TREM2 antibody solution (R&D, AF1729, 1:300). Fibrillar Aβ plaque staining used free-floating sections from 5xFAD mouse cohorts. Sections were permeabilized with 0.25% Triton X-100 in PBS and stained with 10 µM X34 (Sigma, SML1953) in a mixture of 40% ethanol and 0.02 M NaOH in PBS [31]. Following X34 staining, fluorescence immunostaining targeting proteins of interest were performed on the same slice.

To quantify signals of Aβ, X34, IBA1, LAMP1, APP, IBA1, CD68, GFAP and TREM2, images were taken, and stitched using Keyence (BZ-X800) at 20X. For each staining, the hippocampus and the cortex region located above the hippocampus were traced and saved as region of interest (ROI) images in Image J. A unified intensity threshold was applied to all the sample images in each staining. Pixels with the signal intensity above the threshold were used to calculate area percentages of positive immunoreactive signals in ROI through the particle analysis plugin. For Aβ and X34 staining, plaque numbers and sizes were also assessed. Plaque densities were calculated using plaque (diameter > 8 µm [32, 33]) numbers divided by the ROI areas. For IBA1 staining in the non-amyloid mice, a unified intensity threshold was applied to recognize the microglial cell body followed by particle analysis to examine the microglial density and cell body size. To further assess microglial morphology, 4–5 fields were taken per sample under confocal (Zeiss) at 20 × with a zoom factor 0.6. Images were processed to remove background and skeletonized followed by analysis of branch number, junction number and total branch length per microglia [34].

To assess the interaction of microglia and plaque, we co-stained X34 and IBA1. 30-40 z-stack images per sample were taken under Confocal (Zeiss) at 40X with a zoom factor 0.6. Plaque-centered ROI was traced with a radius of 30 µm and saved through Image J. The number of microglia surrounding each plaque within the radius of 30 µm were manually counted. Colocalization of IBA1 and X34 was decided through ‘Colocalization Threshold’ analysis in Image J.

All the analyses were conducted in a batch mode through customized macro coding in Image J and *MATLAB* (2021b) with the same setting parameters for all the samples. Researchers were blinded to genotypes and groups when performing and quantifying the immunofluorescence staining.

### Aβ40, Aβ42, Aβ oligomer, sAPPα, sAPPβ, CTFβ, TNFα*,* and TREM2 ELISA

Aβ40 and Aβ42 levels in TBS, TBSX, and GND lysates were determined by ELISA as previously described [35] using an end-specific Aβ monoclonal antibody (13.1.1 for Aβ40 and 2.1.3 for Aβ42) and an HRP-conjugated detection antibody (in-house Ab5 antibody) [36]. Aβ42 in ISF was detected by commercial kits (Thermo Fisher, KHB3544). Aβ42 oligomers in TBS and TBSX lysates were detected by commercial kits (Biosensis, BEK-2215-2P). Soluble APPα (sAPPα), sAPPβ in TBS lysates were detected by commercial kits (Meso Scale Discovery, K15120E-2). CTFβ in TBSX lysates was detected by commercial kit (IBL, 27776). TNFα was measured in TBS lysates using commercial kit (Meso Scale Discovery, K152QWD-1).

TREM2 in cortical TBS, TBSX lysates, conditioned medium and cell lysates of primary microglia, serum were measured as described [30, 37] with minor modification using the Meso Scale Discovery (MSD) platform. Streptavidin-coated 96-well plates (MSD, L55SA) were blocked overnight at 4 °C in blocking buffer (3% bovine serum albumin and 0.05% Tween-20 in PBS). On the second day, capture antibody (R&D Systems, BAF1729, 0.25 ug/ml) was applied for an incubation at RT for 1 h. After washing with PBST (0.05% Tween-20 in PBS), samples were incubated overnight at 4 °C with an established dilution in fresh-prepared sample buffer (1% bovine serum albumin and 0.05% Tween-20 in PBS) supplemented with protease inhibitor (cOmplete, Roche). Following another wash with PBST, detection antibody (R&D Systems, MAB1729,) was applied for an incubation at RT for 1 h. Sulfo-tag labeled anti rat antibody (MSD, R32AH-5) was applied at RT for 1 h, and final measurements were made with Read Buffer (MSD, R92TC-3). TBS lysate, TBSX lysate, and serum from *Trem2*-KO mice were used as negative controls.

### Parallel reaction monitoring-based targeted quantitation of TREM2 in mouse brain

Mouse brain tissues were snap-frozen immediately after collection. Brain tissues (2 mg) were lysed in lysis buffer (50 mM Tris, pH 7.4, 150 mM NaCl, 1% n-Octylglucoside, HALT protease/phosphatase inhibitor cocktail) by sonication. After centrifugation, the supernatant was transferred as brain lysate and subjected to the following procedures. The protein concentration from the brain lysate was determined by BCA assay. Biotinylated anti-mouse TREM2 antibody (R&D, BAF1729) was bound to Streptavidin-Dynabeads (Life technologies, 29200) by mixing for 30 min at RT. After washing to remove unbound antibody, equal amounts of protein from each sample were incubated with biotinylated anti-mouse TREM2 antibody (R&D, BAF1729) bound to Streptavidin-Dynabeads overnight at 4 °C. The automated KingFish Flex System (ThermoFisher Scientific) was used to wash beads with ice-cold PBS and elute of TREM2 with 5% acetic acid with high reproducibility. The eluents containing TREM2 were subjected to in-solution trypsin digestion. The resulting peptides were spiked with 5 femtomoles (fmol) of stable isotope-labeled internal standard (SIL-IS) peptides unique to sTREM2-WT, flTREM2-WT, sTREM2-H157Y, and flTREM2-H157Y (synthesized by the Mayo Clinic Proteomics Core). Parallel reaction monitoring (PRM) analysis was performed on an Orbitrap Exploris 480 mass spectrometer coupled to Ultimate 3000RSLC NANO LC System (ThermoFisher). The acquired mass spectra were analyzed using Skyline [38–40] to calculate the absolute molar amount of sTREM2 and flTREM2 using the relative ratio of native peptides to SIL-IS peptides.

### Western blotting

Equal amounts of protein from the brain TBS and TBSX lysates, or RIPA lysates from isolated microglia were resolved by SDS-PAGE and transferred to PVDF membranes. After blocking, proteins of interest were detected with appropriate primary antibodies. The membrane was then probed with HRP-conjugated or LI-COR secondary antibodies and visualized using the films or Odyssey infrared imaging system (LI-COR). We used the following primary antibodies against: TREM2 (5F4, ordered from Dr. Haass lab), GLUR2 (Millipore, MAB397, 1:1000), PSD95 (Cell Signaling, 3450 s, 1:1000), synaptophysin (Biolegend, 807801, 1:1000), SYK (Cell Signaling, 2712S, 1:1000), pSYK (Cell Signaling, 2710S, 1:1000) and β-actin (Sigma, A2228, 1:2000).

### RNA extraction, library preparation and sequencing

RNA from pulverized cortex was extracted and purified according to our previous study [41]. The RNA integrity numbers (RIN) of all the 40 RNA sequencing (RNAseq) samples were above 9.5. Thus, they were all used for library preparation and sequencing. RNA libraries were prepared from 200 ng of total RNA using the TruSeq RNA Sample Prep Kit (Illumina) according to the manufacturer’s instructions, employing poly-A mRNA enrichment using oligo-dT magnetic beads. The final adaptor-modified cDNA fragments were enriched by 15 cycles of PCR using Illumina TruSeq PCR primers. The concentration and size distribution of the completed libraries were determined using an Agilent Tape Station, (Agilent) and Qubit fluorometer (Invitrogen). Libraries from all the 40 samples were sequenced on Illumina’s NovaSeq 6000 at 75 million fragment reads/sample following Illumina’s standard protocol on a S2 flow cell. S2 flow cells were sequenced at 100 × 2 paired end reads using a NovaSeq S2 sequencing kit, NovaSeq Control Software v1.7.5 and base-calling was analyzed using Illumina’s RTA version 3.4.4.

### RNAseq data analysis

#### RNA quantification, quality control and normalization

RNA sequencing reads were processed through the Mayo Clinic RNA sequencing analytic pipeline, MAP-RSeq Version 2.1.1 [42]. Briefly, reads were aligned to the mouse reference genome mm10 using TopHat version 2.1.0 [43] and Bowtie version 1.1.2 [44]. Quality control (QC) was performed using RSeQC version 2.6.2 [45]. Gene counts were generated using featureCounts version 1.4.6-p5 [46]. One *Trem2* H157Y (5xFAD) homozygous female sample was excluded from further analysis due to low gene count percentage (39%) and strand-ness check (0.5009). Genes were filtered out from further analyses if there were not at least four samples with 10 counts of the gene. Trimmed Mean of M-values (TMM) normalization was performed with calcNormFactors from the edgeR R package [47].

### Differential gene expression, hierarchical clustering and pathway analysis

Differential gene expression analyses were performed in the comparison of Hom vs WT (separately for non-amyloid or amyloid cohorts) using the edgeR quasi-likelihood pipeline [47]. Differentially expressed genes (DEG) were defined with false discovery rate (FDR) < 0.05 and log_2_|fold change (FC)|> 0.25. Hierarchical clustering was performed in MATLAB using the *Clustergram* function based on standardized Euclidean distance metric. Volcano plots were generated in MATLAB using –log10 (FDR) as y axis and ± log2 (|FC|) as x axis. Pathway analyses of differentially expressed genes were performed through Ingenuity Pathway Analysis (QIAGEN Inc., https://digitalinsights.qiagen.com/products/ingenuity-pathwayanalysis) [48].

### Weighted gene co-expression network analysis and module preservation analysis of the amyloid modules in non-amyloid network

Weighted gene co-expression network analysis (WGCNA) was conducted in non-amyloid mice and amyloid mice, respectively, using residual expression values calculated from adjusting for sex, strand, and exonic rate. Based on the relationship between power and scale independence, the power of 12 was chosen to build scale-free topology using signed hybrid network. We set the minimum modules size as 40 and merged modules whose correlation coefficients were greater than 0.6 (mergeCutHeight = 0.4). Each module was summarized by the first principal component of the scaled module expression profiles, termed module eigengene (ME). For each module, the module membership (MM) was defined as the correlation between gene expression values and ME. Intramodular hub genes are genes with the highest connectivity to other genes within a given module, and were selected based on the p values of MM. To assess the correlation of modules to genotype, we defined the WT genotype as 0 and Hom as 1. Modules were annotated using R package anRichment. MEs of selected modules were compared between genotypes through Wilcoxon rank-sum test. Gene–gene connections among top hub genes were visualized using Cytoscape. GO term enrichment analysis was conducted with anRichment R package.

The preservation of the modules in amyloid network was tested in the non-amyloid network. Separate module preservation analyses were performed for the two datasets using WGCNA. In all analyses, module definitions from the mouse network were used as reference to calculate the z-summary statistics for each module. Z summary score > 2 suggests moderate preservation and Z summary score > 10 suggests strong preservation.

### Quantitative PCR (qPCR)

Purified RNA (1 µg) was used to prepare cDNA iScript™ Reverse Transcription Supermix (Biorad) and qPCR was performed using the QuantStudioTM 7 Flex Real-Time PCR System (ThermoFisher Scientific). To assess the total *Trem2* mRNA, predesigned primers (IDT) targeting exon 4–5 (Mm.PT.58.45957937.g), and customized primers targeting exon 2 (Forward 5′-GCCCATGCCAGCGTGTGGT-3′ and Reverse 5′-CACTGGTAGAGGCCCGC-3′) were used, respectively. Predesigned primers from IDT were used to quantify the mRNA levels of *Tyrobp* (Mm.PT.58.6069426), *Tmem119* (Mm.PT.58.6766267), *Cx3cr1* (Mm.PT.58.17555544), *C1qa* (Mm.PT.58.5375735). The relative gene expression was normalized to *Gapdh* (IDT, Mm.PT.39a.1) and assessed using the 2^−ΔCT^ method.

### In vivo microdialysis

To assess the Aβ clearance, we examined the Aβ level in hippocampal interstitial fluid (ISF) obtained through in vivo microdialysis in awake, free-moving mice as previously described [30, 49, 50]. Animals were placed in a stereotaxic device equipped with dual manipulator arms and an isoflurane anesthetic mask (David Kopf Instruments). Under isoflurane volatile anesthetic, guide cannula (BR style; Bioanalytical Systems) were cemented into the hippocampus (3.1 mm behind bregma, 2.5 mm lateral to midline, and 1.2 mm below dura at a 12˚ angle). Four to six hours post-surgery, a microdialysis probe (30-kilodalton MWCO membrane, Bioanalytical Systems) was inserted through the guide cannula into the brain. Artificial cerebrospinal fluid (aCSF) (mM: 1.3 CaCl_2_, 1.2 MgSO_4_, 3 KCl, 0.4 KH_2_PO_4_, 25 NaHCO_3_, and 122 NaCl, pH 7.4) containing 3% bovine serum albumin (BSA; Sigma) filtered through a 0.1 mm membrane was used as microdialysis perfusion buffer. Flow rate was a constant 1.0 ml/min. Samples were collected hourly into a refrigerated fraction collector. The baseline samples were collected for 10 h followed by subcutaneous administration of a $\gamma$-secretase inhibitor, LY411575 (5 mg/kg) to rapidly block the production of Aβ. Samples were collected for another 4 h after treatment. ISF Aβ42 in the 14 samples for each mouse was measured by ELISA (Invitrogen, KHB3441, 1:4). To determine Aβ42 half-life [49], datapoints from drug delivery were analyzed. Meeting with the first-order processes, the elimination rate ($Ke$) of Aβ42 is related to the slope ($a$) of the semi-log plot of concentration versus time: $a=-Ke/2.3$. The half-life (T_1/2_) of Aβ42 is further calculated as T_1/2_$=0.693/Ke$.

### Statistical analyses

All data were reported as mean values ± SEM. Generally, if sample sizes are larger than 7, to ensure that results were valid in the presence of non-normal distributions, or differing variances between groups, Kruskal–Wallis tests with uncorrected Dun’s multiple comparisons or Wilcoxon Rank-sum tests were used. If the sample size ≤ 7 and dataset showed similar variances examined by F-test, unpaired *t* test was used since nonparametric tests would have very low power. Specifically, One-Way ANOCOVA with comparison of slopes was used in Fig. 2A. Unpaired *t* test with Welch’s correction (Welch’s *t* test) was used Fig. 3Q because of the significantly different variances. Wilcoxon matched-pairs signed rank test was applied to Fig. 6B. All the statistical analyses were conducted using GraphPad Prism v8.4.3 or *MATLAB*. The statistical tests used for each analysis, the sample size and the significance levels were reported in the legend of each figure.

## Results

### Generation of *Trem2* H157Y knock-in mouse model

**Fig. 1:**
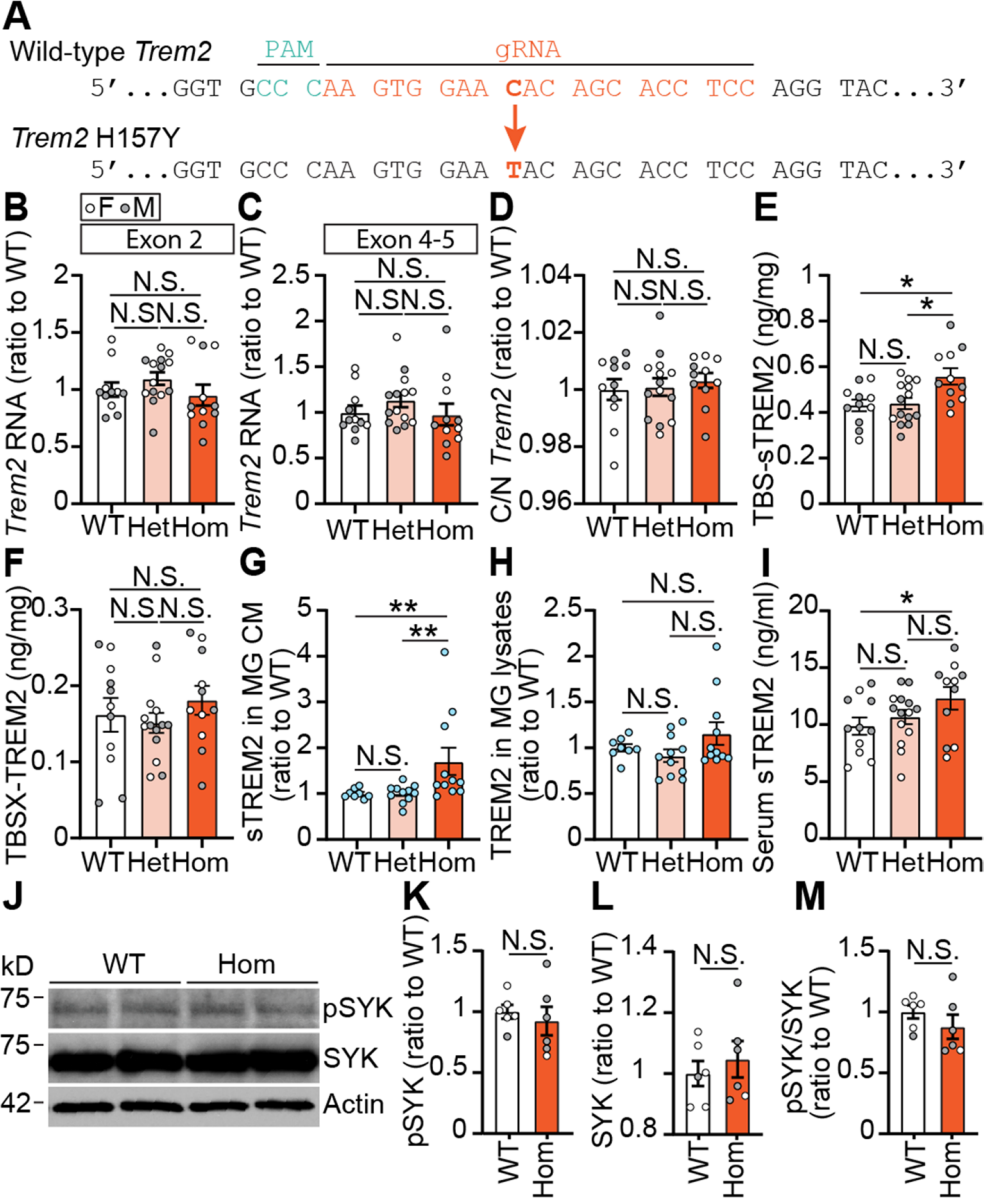
*Trem2* H157Y increases TREM2 shedding. ***A***. *Trem2* H157Y knock-in mice were generated by introducing a C > T mutation (bold orange) via CRIPR/Cas9. Protospacer region recognized by guide RNA (gRNA) is shown in orange. Protospacer adjacent region (PAM) is indicated in green. ***B-C***. Cortical *Trem2* mRNA levels were examined using primers targeting exon 2 (N-terminal, ***B***) or exon 4–5 (C-terminal, ***C***), and normalized to WT mice for each genotype. ***D***. Cycle threshold ratios of C-terminal *Trem2* to N-terminal *Trem2* (C/N *Trem2*) were calculated and normalized to WT mice for each genotype. ***E–F***. TREM2 levels were examined by ELISA and normalized to WT mice in cortical TBS (***E***) and TBSX (***F***) lysates for each genotype. ***G-H***. TREM2 levels were examined by ELISA in conditioned medium (CM) (***G***) and RIPA lysates (***H***) of primary microglia (MG). TREM2 amount was normalized to the total protein level of cell lysates followed by another normalization to WT littermates. *N* = 8–11 pups per genotype. Unknown sex of each pup. ***I***. Serum TREM2 were examined by ELISA in mice from each genotype. ***J***. SYK, phosphorylated SYK (pSYK) and actin were detected in the RIPA lysates of isolated microglia from WT and Hom mice. ***K-L***. pSYK (***K***) and SYK (***L***) were quantified and normalized to WT. ***M***. Ratios of pSYK/SYK were calculated and normalized to the WT mice. ***B-F***, ***I***. *N* = 11–14 mice per genotype at 6 months of age, mixed sex. Kruskal–Wallis tests with uncorrected Dun’s multiple comparisons were used in ***B-I***. ***J-M***. *N* = 6 mice/genotype at 6 months of age, mixed sex. Unpaired t-tests were used. Data are presented as Mean ± SEM. N.S., not significant. * *p* < 0.05. **p < 0.01

TREM2-H157 is located where TREM2 undergoes proteolytic shedding to produce sTREM2 [22–24]. To study the in vivo effects of the *Trem2* H157Y mutation, we introduced a C > T substitution in exon3 through CRISPR/Cas9 technology to create the missense H157Y mutation (Fig. 1A). Two founders (1 and 2) were obtained with no off-target mutation observed in the offspring from either founder (Fig. S1A-B). Results reported below were generated using the offspring of founder 1 unless otherwise stated. By crossing the *Trem2* H157Y heterozygous mice, we obtained three genotypes: wild type (*Trem2*^+*/*+^, referred to as WT), heterozygous (*Trem2*^+*/H157Y*^, referred to as Het), and homozygous (*Trem2*^*H157Y/H157Y*^, referred to as Hom). Littermates of these three genotypes were used to investigate the impacts of the *Trem2* H157Y mutation on TREM2 proteolytic processing, microglial density and morphology, synaptic plasticity, and cognitive function.

It was reported that *Trem2* R47H knock-in mouse models generated through CRISPR/Cas9 technologies exhibit aberrant splicing and reduced expression levels of *Trem2* mRNA [51]. We therefore assessed how *Trem2* H157Y affects *Trem2* transcription. We measured total *Trem2* mRNA using primers either targeting the N-terminal (exon 2) or C-terminal part (exon 4–5) of *Trem2* in mice of each genotype at 6 months of age. Neither of these primers recognized significant differences of total *Trem2* level between genotypes (Fig. 1B-C). In addition, we calculated the ratios of cycle thresholds (Ct) for the C-terminal and N-terminal *Trem2* (C/N *Trem2*) and did not observe significant differences of these ratios between genotypes (Fig. 1D), suggesting no aberrant splicing of *Trem2* in our *Trem2* H157Y knock-in mouse model.

### *Trem2* H157Y increases the production of sTREM2

We proceeded to evaluate TREM2 protein levels in each genotype. Proteins were sequentially extracted from cortex with Tris-buffered saline (TBS) and TBSX (TBS + 1% Triton X-100) and analyzed by N-terminal TREM2-capturing ELISA. Although membrane bound TREM2 in TBSX did not differ between genotypes (Fig. 1F), there was an increase of sTREM2 in the TBS lysates in Hom compared to Het and WT mice (Fig. 1E). To further examine the effects of *Trem2* H157Y on TREM2 proteolytic processing in microglia, we cultured cortical primary microglia from littermate pups of our mouse model. Consistent with in vivo findings, we observed an increase of sTREM2 in conditioned medium (CM) from Hom microglia compared to that from Het and WT microglia (Fig. 1G). The membrane associated TREM2 in microglia RIPA lysates did not differ between genotypes (Fig. 1H). Further supporting an increase of sTREM2 production in the presence of the *Trem2* H157Y mutation, we observed higher levels of serum sTREM2 in Hom mice compared to WT mice (Fig. 1I).

To examine the TREM2 signaling potentially impacted by *Trem2* H157Y, we isolated microglia from WT and Hom mice at 6 months of age and lysed them in the RIPA buffer. Through western blotting, we quantified the spleen tyrosine kinase (SYK), phosphorylated SYK (pSYK) (Fig. 1J) and calculated the pSYK/SYK ratios. No significant differences were observed between WT and Hom mice in the levels of pSYK (Fig. 1K) and total SYK (Fig. 1L), as well as pSYK/SYK ratios (Fig. 1M), indicating limited impact of *Trem2* H157Y on TREM2 signaling.

Collectively, our results support that the *Trem2* H157Y mutation increases sTREM2 production without significantly affecting TREM2 signaling in the homozygous mice.

### *Trem2* H157Y does not affect microglia density and morphology

To quantify the microglia density and assess the morphology of microglia, we performed IBA1 immunofluorescence staining of brain slices from *Trem2* H157Y knock-in mice at 6 months of age. Microglia density and cell body size did not change with the *Trem2* H157Y mutation (Fig. S2A-C). Analyses after microglia skeletonization (Fig. S2D-F) showed no significant differences in the branch number, junction number, and total branch length per microglia between genotypes (Fig. S2G-I). These results suggest *Trem2* H157Y does not affect microglia density and morphology in vivo under physiological conditions.

### *Trem2* H157Y enhances synaptic plasticity

*Trem2* deficiency, *Trem2* R47H, *Trem2* Y38C have been reported to affect synaptic activities [52–56]. We examined the levels of synaptic markers in mice of each genotype at 6 months of age through Western blotting. No significant differences were detected between genotypes in synaptophysin, PSD95, and GLUR2 levels, indicating the synaptic integrity is unaffected by *Trem2* H157Y (Fig S3A-D).

**Fig. 2:**
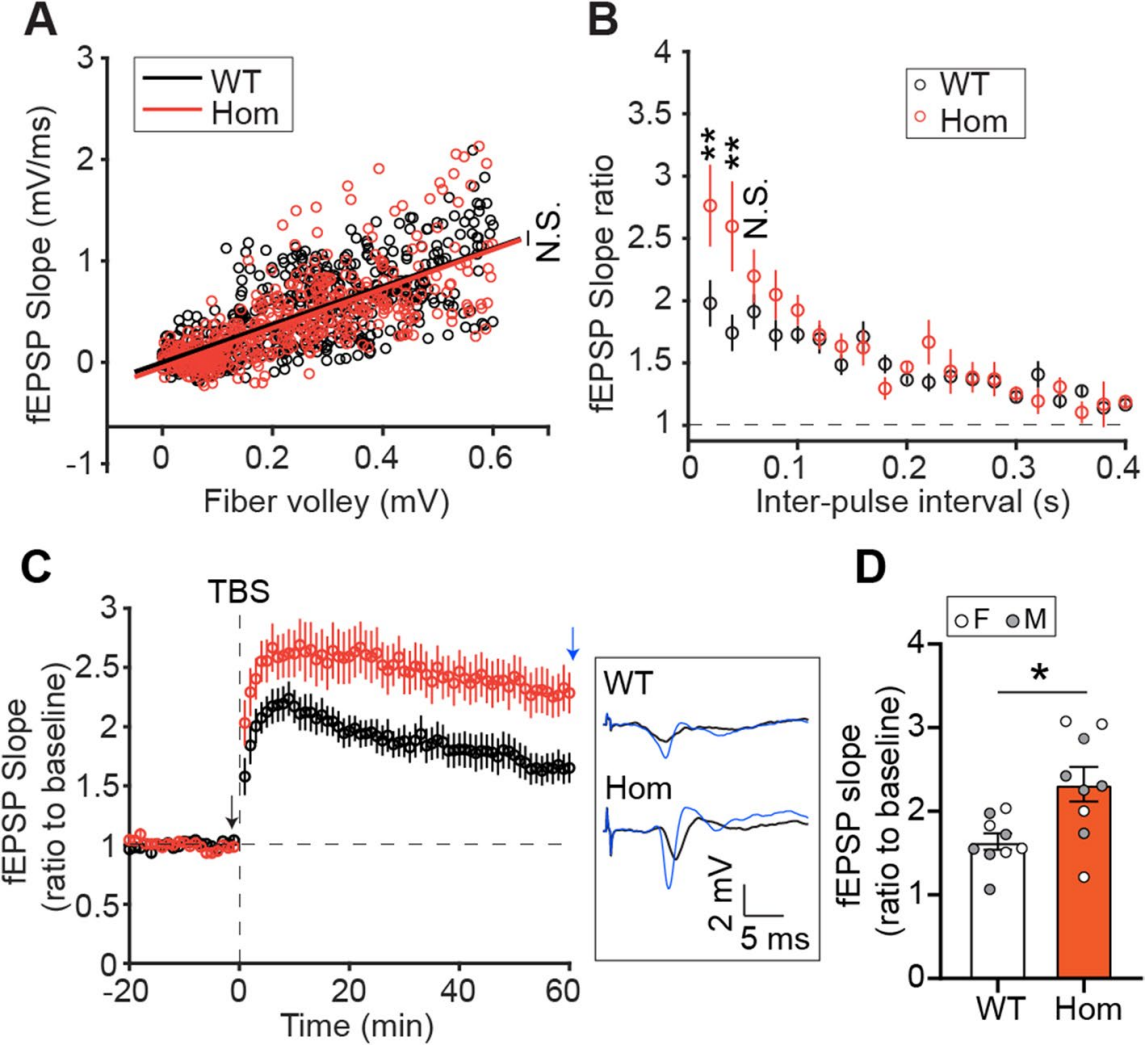
*Trem2* H157Y enhances synaptic plasticity. ***A***. The input–output curves for WT and Hom mice at 6 months of age are shown as linear regressions of fEPSP slopes corresponding to pre-synaptic fiber volley amplitudes. WT, n/N = 21 brain slices from 9 mice; Hom, n/N = 18/8; mixed sex. ***B.*** Paired-pulse facilitation profiles are shown. Ratios of fEPSP slopes evoked by second pulse and first pulse were calculated and plotted versus the corresponding inter-pulse intervals. WT, n/N = 17/8; Hom, n/N = 14/7; mixed sex. ***C***. Theta-Burst Stimulus (TBS) induced LTP profiles for WT and Hom mice are shown as averaged and normalized fEPSP slopes. Example baseline (black arrow, black trace) and last-minute (blue arrow, black trace) fEPSP traces are shown in the inset frame. ***D.*** The average fEPSP slopes in the last five minutes were compared between WT and Hom mice. WT, n/N = 20/9; Hom, n/N = 16/9; mixed sex. Data are presented as Mean ± SEM. One-Way ANOCOVA with comparison of slopes was used in ***A***. Wilcoxon Rank-sum tests were used in ***B*** and*** D***. N.S., not significant. *p < 0.05. **p < 0.01

We then performed hippocampal long-term potentiation (LTP) recording in WT and Hom mice of our mouse model at 6 months of age to examine whether the *Trem2* H157Y mutation affects synaptic function. Similar slopes of input–output curves from WT and Hom mice were observed suggesting the *Trem2* H157Y mutation does not affect the basic transmission (Fig. 2A). However, stronger paired-pulse facilitation was observed in Hom mice compared to WT mice (Fig. 2B) suggesting the presynaptic function and short-term plasticity are enhanced in the Hom mice. Moreover, Hom mice showed strengthened LTP compared to WT mice (Fig. 2C and D) using a theta-burst stimulation paradigm indicating the synaptic plasticity is enhanced.

To examine whether this strengthened synaptic capability with *Trem2* H157Y leads to spatial cognitive performance, we conducted a battery of behavioral tests with mice of each genotype at 6 months of age. We did not observe significant performance differences in anxiety (Fig. S3E) and associative memory assessments (Fig. S3G-H) between genotypes. However, using Y-maze spontaneous alteration tests, we observed a trending performance improvement of spatial working memory in Hom mice compared to Het mice while no difference between Het mice and WT mice (Fig. S3F; Het vs Hom, *p* = 0.07).

Taken together, these results support a beneficial effect of *Trem2* H157Y on synaptic plasticity and presynaptic function, even though it did not translate into significant enhancement at the behavioral level in our paradigms.

### *Trem2* H157Y does not affect amyloid pathology at the early stage of amyloid development

To investigate the effects of H157Y mutation on AD-related amyloid pathogenesis, we crossed *Trem2* H157Y knock-in mice with 5xFAD amyloid model mice. Early-stage amyloid pathologies were assessed in mice of each genotype at 4 months of age [57] through Aβ immunostaining of brain slices with MOAB2 antibody (Fig. S4A) and fibrillar Aβ stain with X34 (Fig. S4B). No significant differences between genotypes were observed in plaque coverages (Fig. S4C, F, I and L), densities (Fig. S4D, G, J and M), and sizes (Fig. S4E, H, K and N) in the cortex (Fig. S4C-E, I-K) or hippocampus (Fig. S4F-H, L-N). We also quantified the levels of Aβ40 and Aβ42 by ELISA in different cortical lysates which were obtained through sequential protein extraction with TBS, TBSX, and guanidine (GND) buffer. No significant differences between genotypes were observed in the measurements of Aβ40 and Aβ42 in TBS (Fig. S4O-P), TBSX (Fig. S4Q-R) and GND lysates (Fig. S4S-T).

We also assessed the microgliosis and astrogliosis in response to the amyloid pathology, through IBA1 (Fig. S5A) and GFAP (Fig. S5D) immunostaining, respectively. Hom mice exhibited a trending of lower IBA1^+^ areas in the cortex (Fig. S5B, Hom vs WT, *p* = 0.06) and hippocampus (Fig. S5C, Hom vs WT, *p* = 0.09) compared to WT mice. No significant differences of IBA1^+^ areas were observed between Het and WT mice. On the other hand, cortical and hippocampal GFAP^+^ areas showed no significant differences between genotypes, indicating the astrogliosis is not affected in our animal model.

Taken together, these results suggest that *Trem2* H157Y does not affect early-stage amyloid pathologies.

### *Trem2* H157Y reduces amyloid burden in 5xFAD mice at the late stage of amyloid development

**Fig. 3:**
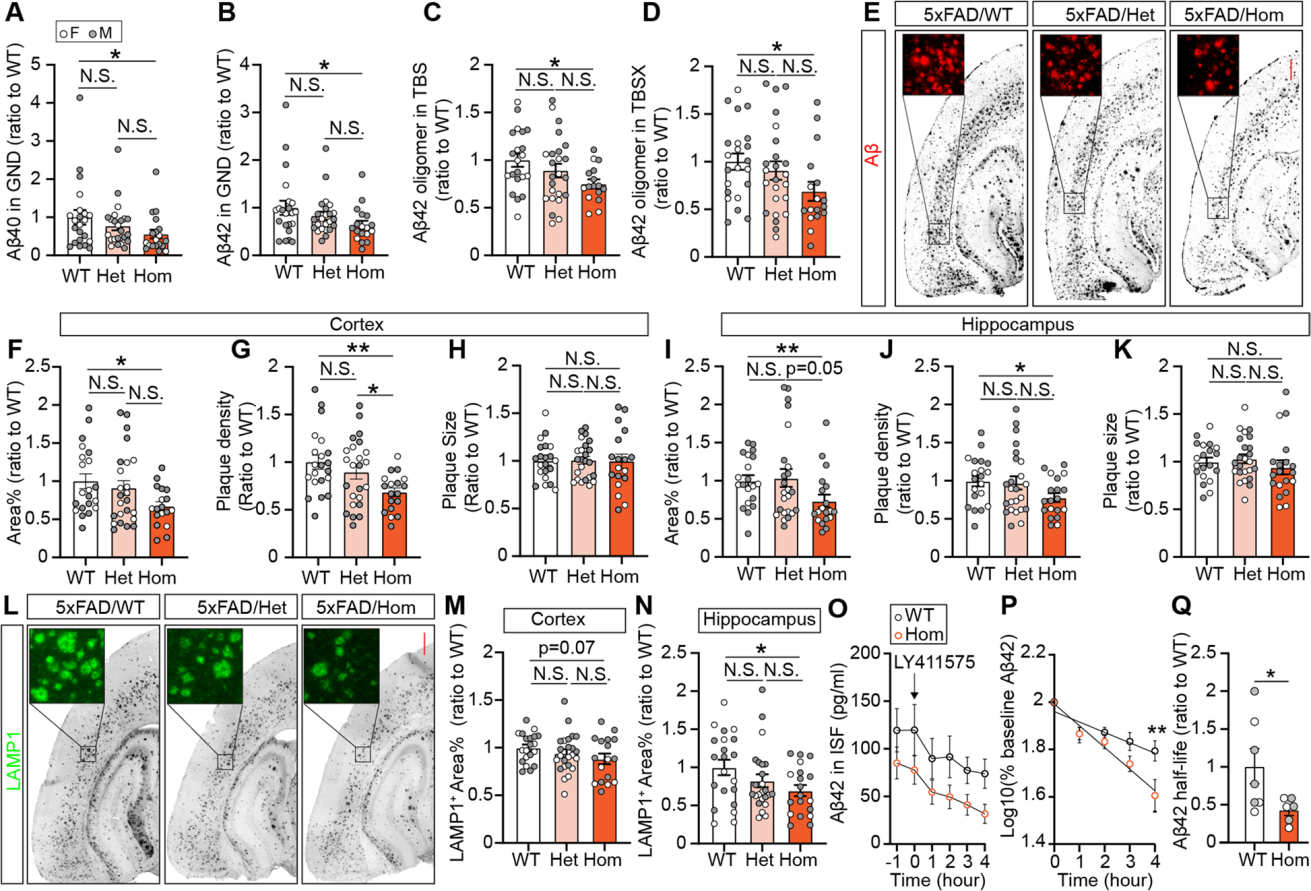
*Trem2* H157Y reduces amyloid burden and dystrophic neurites in 5xFAD mice at 8.5 months of age. *A-B*. Aβ40 (***A***) and Aβ42 (***B***) were quantified by ELISA and normalized to WT in cortical guanidine lysates (GND) for each genotype. ***C-D***. Aβ42 oligomer were quantified by ELISA and normalized to WT in cortical TBS (***C***) and TBSX (***D***) lysates for each genotype. ***E***. Representative images of amyloid (MOAB2) staining are shown for each genotype. Scale, 400 µm. ***F-K***. Cortical (***F–H***) and hippocampal (***I-K***) amyloid plaque area coverages (***F, I***), densities (***G, J***) and sizes (***H, K***) were quantified and normalized to WT mice. ***L***. Representative images of LAMP1 staining for dystrophic neurites are shown. Scale, 400 µm. ***M–N***. Cortical (***M***) and hippocampal (***N***) LAMP1^+^ area coverages were quantified and normalized to WT mice for each genotype. ***A-N***. *N* = 19–24 mice per genotype at 8.5 months of age, mixed sex. Data are presented as Mean ± SEM. Kruskal–Wallis tests with uncorrected Dun’s multiple comparisons were used. ***O***. Aβ42 was quantified by ELISA in the interstitial fluid (ISF) obtained in microdialysis experiments with WT and Hom mice at 3 months of age. At time 0, γ-secretase inhibitor LY411575 was administrated to stop the Aβ production. ***P***. Semilog plot was performed from time 0 to analyze the half-life of Aβ42 clearance. ***Q***. Half-life was calculated and plotted in WT and Hom mice with a normalization to WT mice. ***O-Q***. *N* = 6–7 mice per genotype at 3 months of age, mixed sex. Data are presented as Mean ± SEM. Unpaired *t* tests were used in ***P***. Welch’s *t* test was used in ***Q***. N.S., not significant. * *p* < 0.05. **p < 0.01

Next, we assessed the late-stage amyloid pathology in 5xFAD mice of each genotype at 8.5 months of age [57]. Interestingly, at this age, Hom mice showed significantly lower Aβ40 and Aβ42 in cortical GND lysates compared to WT mice while no significant differences were detected between Het mice and Hom or WT mice (Fig. 3A-B). The levels of Aβ40 and Aβ42 in cortical TBS (Fig. S6A-B) and TBSX lysates (Fig. S6C-D) showed no significant differences between genotypes. However, significantly lower amount of the neuronal toxic species, Aβ42 oligomers were detected in TBS and TBSX lysates of Hom mice compared to WT mice (Fig. 3C-D). Consistent with the above results, Aβ immunostaining with MOAB2 antibody (Fig. 3E) revealed significant reductions of Aβ plaque coverages (Fig. 3F, I) and densities (Fig. 3G, J) in the cortex and hippocampus of Hom mice compared to WT mice. Plaques from all three genotypes were found to be similar in size (Fig. 3H, K). We did not observe significant decreases of X34-positive fibrillar Aβ (Fig. S6E) plaque coverages (Fig. S6F, I), densities (Fig. S6G, J), or sizes (Fig. S6H, K) in the cortex and hippocampus of Hom or Het mice compared to WT mice.

Further, to examine the effects of *Trem2* H157Y on Aβ related neuronal toxicity, we assessed the dystrophic neurites through lysosome-associated membrane protein (LAMP1) immunostaining (Fig. 3L). LAMP1^+^ areas showed a trending decrease in the cortex (Fig. 3M, Hom vs WT, *p* = 0.07) and a significant decrease in the hippocampus (Fig. 3N) of Hom mice compared to WT mice. No significant differences were found between Het and Hom or WT mice. To validate this observation, we stained dystrophic neurites with a C-terminal APP antibody (Fig. S6L). Consistent with the LAMP1 staining result, quantification of APP^+^ areas showed significant decreases in the cortex (Fig. S6M) and hippocampus (Fig. S6N) of Hom mice compared to WT mice. Additionally, we assessed synaptic integrity through detection of PSD95 and synaptophysin by Western blotting in cortical TBSX lysates (Fig. S6O). However, we did not detect significant changes of these synaptic markers in Hom mice compared with WT mice (Fig. S6P-Q), suggesting that there is no significant neuronal/synaptic loss in our animal models with the *Trem2* H157Y mutation.

Taken together, these assessments suggest that *Trem2* H157Y reduced amyloid burden and Aβ related neurite dystrophy in homozygous mice at the late stage of amyloid development.

### *Trem2* H157Y facilitates Aβ clearance in 5xFAD mice

To address the potential mechanism of amyloid reduction in the *Trem2* H157Y homozygous mice, we examined the APP processing products [58] and found no significant differences in the levals of cortical sAPPα, sAPPβ, and CTFβ between genotypes (Fig. S6R-T) at 8.5 months of age, suggesting unaltered Aβ production by the *Trem2* H157Y mutation. We then set out to assess the Aβ clearance affected by *Trem2* H157Y. To avoid the impacts of amyloid plaque on the Aβ clearance [49], we conducted in vivo microdialysis with awake, free-moving mice at 3 months of age [30, 49, 50]. We analyzed Aβ42 clearance in the interstitial fluid (ISF) while Aβ production was inhibited with γ-secretase inhibitor, LY411575 (Fig. 3O). The elimination kinetic analysis showed enhanced clearance of Aβ42 with decreased ISF-Aβ42 levels four hours post drug administration (Fig. 3P) and a 50% reduction of Aβ42 half-life (Fig. 3Q) in Hom mice compared to WT mice. The enhanced clearance of Aβ by *Trem2* H157Y may accumulatively lead to the overall suppression of amyloid pathology in Hom mice at 8.5 months of age.

### *Trem2* H157Y reduces gliosis in 5xFAD mice at the late stage of amyloid development

**Fig. 4:**
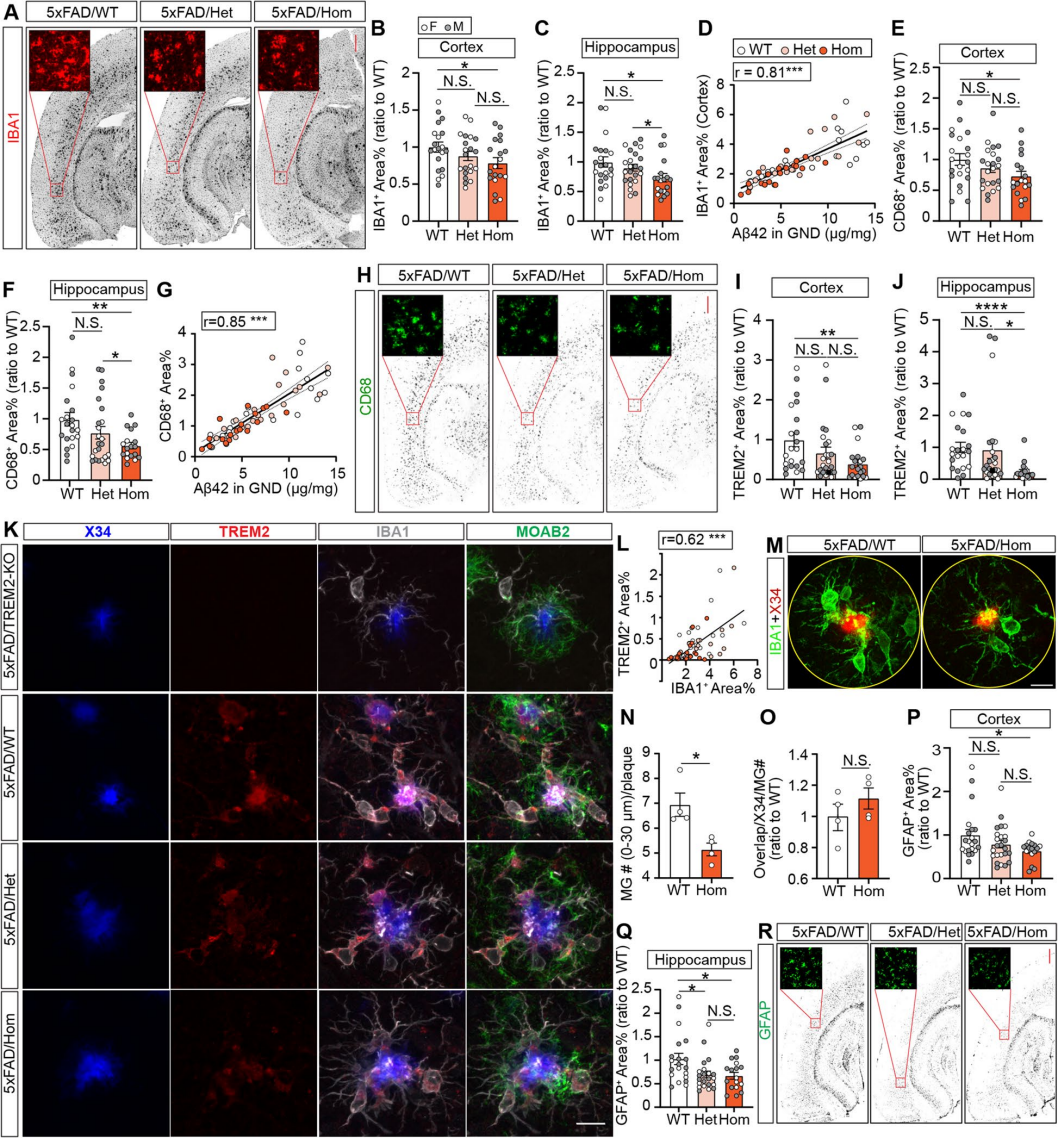
*Trem2* H157Y reduces glial responses in 5xFAD mice at 8.5 months of age. ***A***. Representative images of IBA1 immunostaining are shown for each genotype. Scale, 400 µm. ***B-C***. Cortical (***B***) and hippocampal (***C***) IBA1^+^ area percentages were quantified and normalized to WT. ***D***. The correlation between IBA1^+^ area percentages and Aβ42 in GND lysates is shown with correlation coefficient (r) and significance symbols. ***E–F***. Cortical (***E***) and hippocampal (***F***) CD68^+^ area percentages were quantified for each genotype. ***G***. Correlation between CD68^+^ area percentages and Aβ42 in GND lysates are shown with correlation coefficient (r) and significance symbols. ***H***. Representative images of CD68 immunostaining are shown for each genotype. Scale, 400 µm. ***I-J***. Cortical (***I***) and hippocampal (***J***) TREM2^+^ area coverages were quantified and normalized to WT mice. ***K***, Representative confocal images of X34, TREM2, IBA1 and MOAB2 co-staining are shown for each genotype. Scale, 10 µm. ***L***. The correlation between TREM2^+^ and IBA1^+^ area percentages is shown with correlation coefficient (r) and significance symbols. ***M***. Representative confocal images of IBA1 and X34 co-staining are shown for each genotype. Scale, 10 µm. ***N***. Microglial numbers surrounding X34 signal were counted and quantified within a radius of 30 µm for each genotype. ***O***. Plaque (X34) area coverages by microglia (MG) (IBA1) were quantified, ratioed to microglia number and normalized to WT within a radius of 30 µm for each genotype. ***N–O***. N = 4 mice per genotype at 8.5 months of age. Data are presented as Mean ± SEM. Unpaired t-tests were used. ***P-Q***. Cortical (***P***) and hippocampal (***Q***) GFAP^+^ area percentages were quantified and normalized to WT mice. ***R***. Representative images of GFAP immunostaining are shown for each genotype at 8.5 months of age. Scale, 400 µm. ***A-L, P-R***. *N* = 19–24 mice per genotype at 8.5 months of age, mixed sex. Data are presented as Mean ± SEM. Kruskal–Wallis tests with uncorrected Dun’s multiple comparisons were used in ***B, C, E***, ***F***,*** I***,*** J***, ***P*** and*** Q***. N.S., not significant. *p < 0.05. **p < 0.01. ***p < 0.001. ****p < 0.0001

To assess the microglial responses to amyloid pathology with *Trem2* H157Y, we performed immunostaining of microglial marker IBA1 (Fig. 4A) and the phagocytic marker CD68 (Fig. 4H) with brain slices from mice at 8.5 months of age. Significant reductions of IBA1^+^ and CD68^+^ areas were observed in the cortex (Fig. 4B, E) and hippocampus (Fig. 4C, F) of Hom mice compared to WT, suggesting reduced microgliosis and neuroinflammation with *Trem2* H157Y. No significant differences were detected between Het mice and WT or Hom mice. We further found positive correlations between IBA1^+^ or CD68^+^ areas and GND-Aβ42, suggesting that the decreased microgliosis is likely related to the amyloid pathology (Fig. 4D and G). We also stained TREM2 in brain slices of each genotype (Fig. 4K) and observed significant reductions of TREM2^+^ areas in Hom mice compared to WT mice in the cortex (Fig. 4I) and hippocampus (Fig. 4J). Positive correlation was further found between TREM2^+^ and IBA1^+^ areas (Fig. 4L), suggesting the reduction of TREM2 immunoreactivity may result from decreased microgliosis in Hom mice.

Plaque-associated microglia have been identified as a critical pathological event in response to amyloid [2]. We therefore quantified the number of microglia associated with amyloid plaques (Fig. 4M and N) in WT and Hom mice but not in Het mice given that they did not show significant changes in the amyloid load and microgliosis compared to WT and Hom mice. A decrease of microglia number per plaque was found in a plaque-centered radius of 30 µm in Hom mice compared to WT mice (Fig. 4N). However, there were no significant differences between genotypes in the plaque area coverage by IBA1^+^ signals after a normalization of the coverage to the microglia number (Fig. 4O) suggesting the decreased number of microglia surrounding plaques is a result of the general microgliosis reduction in Hom mice.

We also stained GFAP (Fig. 4R) to assess the astrogliosis and observed reduced GFAP^+^ areas in the cortex (Fig. 4P) and hippocampus (Fig. 4Q) of Hom mice compared to WT mice. Hippocampal GFAP^+^ areas were also significantly reduced in Het mice compared to WT mice (Fig. 4Q). Collectively, we found *Trem2* H157Y reduced gliosis at the late stage of amyloid development.

### *Trem2* H157Y reduces amyloid pathology and gliosis in mice of a different founder line at the late stage of amyloid development

**Fig. 5:**
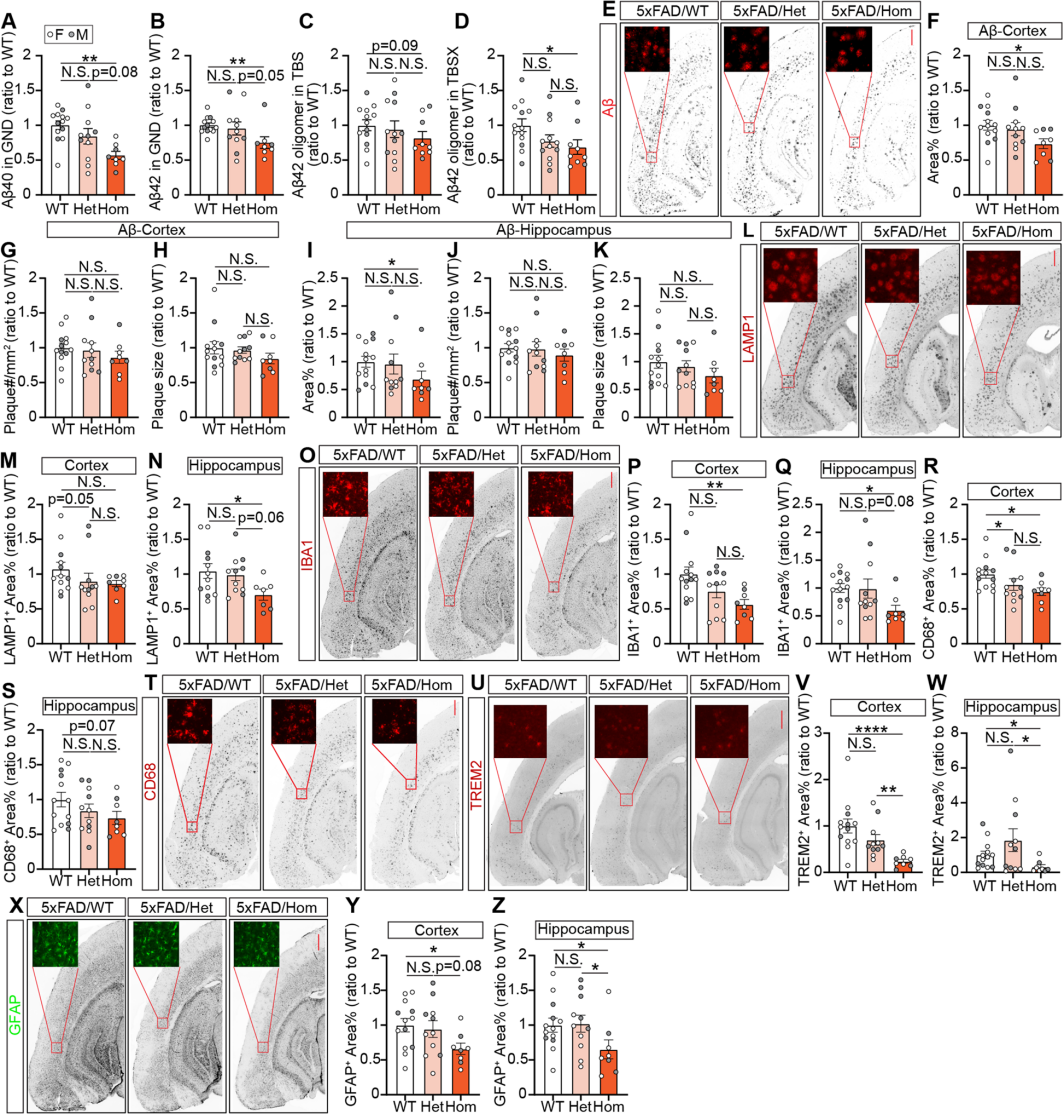
*Trem2* H157Y reduces amyloid pathologies in Founder 2-linage mice at 8.5 months of age. *A-B*. Aβ40 (***A***) and Aβ42 (***B***) were quantified by ELISA and normalized to WT mice in cortical guanidine lysates (GND) for each genotype. ***C-D***. Aβ42 oligomers were quantified by ELISA and normalized to WT mice in cortical TBS (***C***) and TBSX (***D***) lysates. ***E***. Representative images of amyloid staining are shown for each genotype. Scale, 400 µm. ***F-K***. Cortical (***F–H***) and hippocampal (***I-K***) amyloid plaque area percentages (***F, I***), densities (***G, J***) and sizes (***H, K***) are quantified and normalized to WT mice for each genotype. ***L***. Representative images of dystrophic neurite staining (LAMP1^+^) are shown for each genotype. Scale, 400 µm. ***M–N***. Cortical (***M***) and Hippocampal (***N***) LAMP1^+^ area percentages were quantified and normalized to WT mice for each genotype. ***O***. Representative images of IBA1 immunostaining are shown for each genotype. Scale, 400 µm. ***P-Q***. Cortical (***P***) and hippocampal (***Q***) IBA1 + area percentages were quantified for each genotype. ***R-S***. Cortical (***R***) and hippocampal (***S***) CD68^+^ area percentages were quantified and normalized to WT mice for each genotype. ***T***. Representative images of CD68 immunostaining are shown for each genotype. Scale, 400 µm. ***U***. Representative images of TREM2 immunostaining are shown for each genotype. Scale, 400 µm. ***V-W***. Cortical (***V***) and hippocampal (***W***) CD68^+^ area percentages were quantified and normalized to WT mice for each genotype. ***X***. Representative images of GFAP immunostaining are shown for each genotype. Scale, 400 µm. ***Y–Z***. Cortical (***Y***) and hippocampal (***Z***) GFAP^+^ area percentages were quantified and normalized to WT for each genotype. ***A-Z***. N = 8–13 mice per genotype, at 8.5 months of age, mixed sex. Data are presented as Mean ± SEM. Kruskal–Wallis tests with uncorrected Dun’s multiple comparisons were used. N.S., not significant. * *p* < 0.05. **p < 0.01. ****p < 0.0001

We further examined the effects of Trem2 H157Y on the amyloid pathologies in *Trem2* H157Y knock-in mice of a second founder line (founder 2). Through measuring Aβ40 and Aβ42 by ELISA in different cortical lysates (Fig. 5A-B, Fig. S7A-D), Aβ42 oligomers in the TBS and TBSX lysates (Fig. 5C-D), as well as Aβ immunostaining (Fig. 5E-K), we observed significant reductions in the GND-Aβ40 and -Aβ42, TBS-Aβ40 and -Aβ42 (Fig. 5A-B, Fig. S7A-B), TBSX-Aβ42 oligomer (Fig. 5D), and Aβ plaque areas (Fig. 5F-I) in Hom mice compared to WT mice, while the fibrillar Aβ load was unaltered by *Trem2* H157Y through the analysis of X34 staining (Fig. S8E-K). Through LAMP1 (Fig. 5L-N) and C-terminal APP (Fig. S7L-N) staining, we observed significant or trending reductions of dystrophic neurites in the cortex and hippocampus in Hom mice compared to WT mice.

Moreover, through IBA1(Fig. 5O), CD68 (Fig. 5T), TREM2 (Fig. 5U) staining, we observed significant or trending reductions of IBA1^+^ (Fig. 5P-Q), CD68^+^ (Fig. 5R-S), TREM2^+^ (Fig. 5V-W) areas in the cortex and hippocampus of Hom mice compared to WT mice. Through GFAP immunostaining (Fig. 5X), we observed significant reductions of GFAP^+^ areas in the cortex (Fig. 5Y) and hippocampus (Fig. 5Z) of Hom mice compared to WT mice.

Taken together, amyloid pathological analysis in founder 2-linage mice showed consistent results with those from founder 1-linage mice that *Trem2* H157Y reduces amyloid burden, dystrophic neurites, and gliosis at 8.5 months of age.

### *Trem2* H157Y facilitates TREM2 shedding and reduces TREM2 signaling in 5xFAD mice

**Fig. 6:**
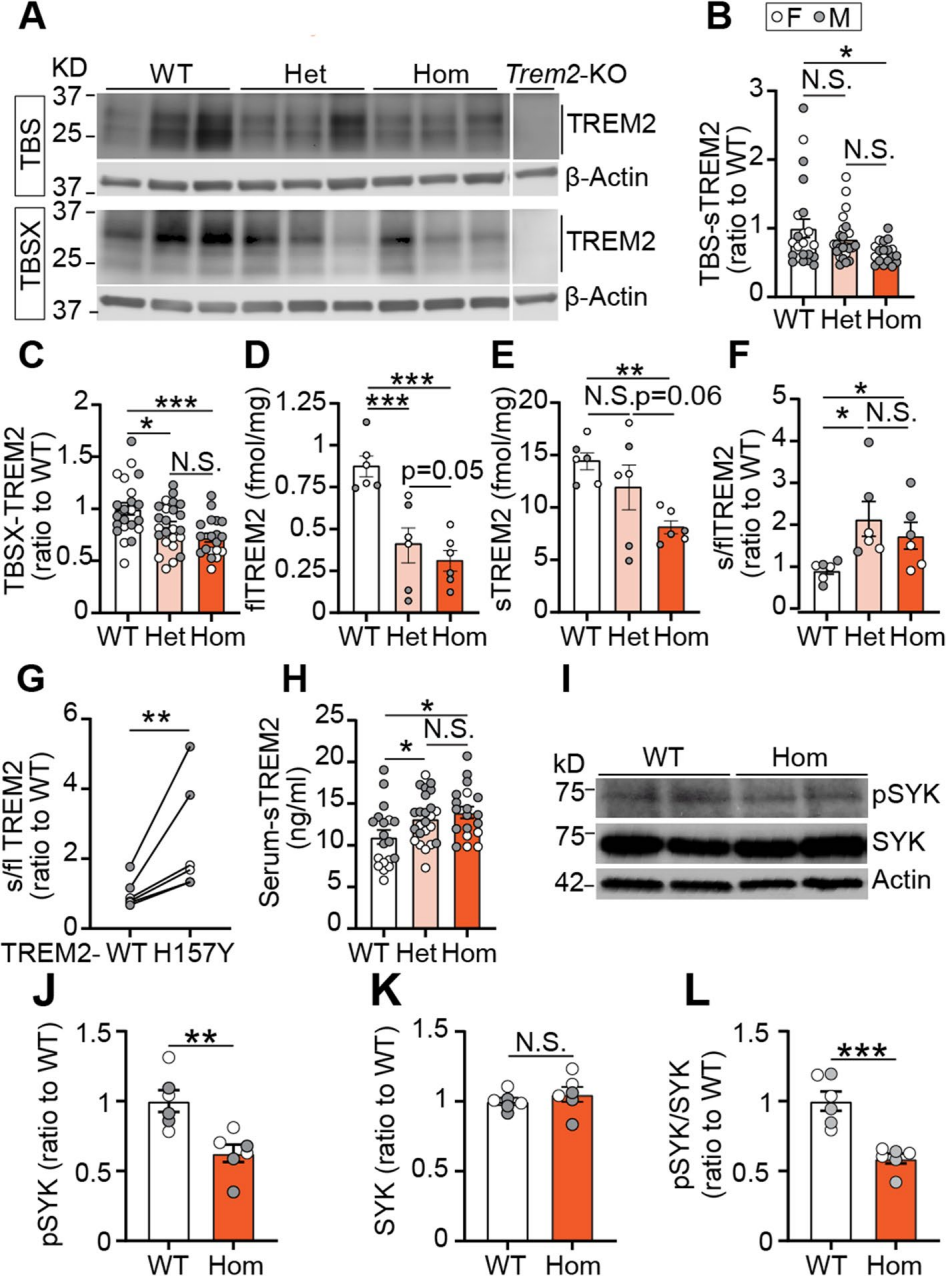
*Trem2* H157Y increases TREM2 shedding in 5xFAD mice. **A**. TREM2 in TBS and TBSX lysates from our mouse models and Trem2-KO mouse were detected using an N-terminal antibody (5F4) through Western blotting. ***B-C.*** TREM2 levels in TBS (**B**) and TBSX (**C**) were quantified and normalized to WT for each genotype. Bands indicated below 37kD were quantified. ***D***. Full-length TREM2 levels were measured in the brain tissues from mice of each genotype. ***E***. Soluble TREM2 levels were measured in the brain tissues from mice of each genotype.*** F***. Ratios of soluble versus full-length (s/fl) TREM2 amount were calculated and normalized to WT mice. ***G***. Ratios of s/fl TREM2 amount were calculated for TREM2-WT and TREM2-H157Y and normalized to TREM2-WT in Het mice. ***D-G***, N = 3 mice/sex/ genotype at 8.5 months of age, mixed sex. Data are presented as Mean ± SEM. One-way ANOVA with multiple comparisons was used in ***D-F*** with Welch's correction. Wilcoxon matched-pairs signed rank test was applied to ***G***. ***H***. Serum TREM2 was examined by ELISA for each genotype. ***I.*** SYK, pSYK and actin were detected in the RIPA lysates of isolated microglia from WT and Hom mice. ***J-K.*** pSYK (***J***) and SYK (**K**) were quantified and normalized to WT. ***L.*** Ratios of pSYK/SYK were calculated and normalized to the WT mice. ***A-C, H***. *N* = 19–24 mice per genotype at 8.5 months of age, mixed sex. Kruskal–Wallis tests with uncorrected Dun’s multiple comparisons were used. ***I-L.****N* = 3 mice/sex/genotype at 8.5 months of age. Unpaired t-tests were used. Data are presented as Mean ± SEM. N.S., not significant. * *p* < 0.05. **p < 0.01. ***p < 0.001

We then set out to evaluate the TREM2 proteolytic processing affected by *Trem2* H157Y in 5xFAD mice at 8.5 months of age. Through Western blotting, we detected TREM2 in TBS and TBSX lysates using an N-terminal TREM2 antibody (5F4) (Fig. 6A). Significant reductions of sTREM2 in TBS lysates (Fig. 6B) and membrane-associated TREM2 in TBSX lysates (Fig. 6C) were observed in Hom mice compared to WT and Het mice while there were no significant differences between WT and Het mice. These results are consistent with the significant reductions of TREM2^+^ areas in Hom mice compared to WT mice found through TREM2 immunostaining (Fig. 4I-K, Fig. 5V-W).

Furthermore, we developed a workflow to enrich TREM2 and measure flTREM2 and sTREM2 (Fig. S8A). Briefly, TREM2 was immunoprecipitated from brain tissue of each genotype with a biotinylated N-terminal TREM2 antibody (R&D, BAF1729) (Fig S8B). After TREM2 immunoprecipitation, the eluate was subjected to targeted mass spectrometry to detect TREM2 after spiking in four peptides (Fig. S8C) derived from flTREM2-WT (Fig. S8D), sTREM2-WT (Fig. S8E), flTREM2-H157Y (Fig. S8F), and sTREM2-H157Y (Fig. S8G), respectively. These peptides permitted quantitation of the four TREM2 types in brain tissues from mice of each genotype (Fig. S8H-K). Further quantification of total amount of flTREM2 demonstrated a significant reduction in Hom and Het mice compared to WT mice (Fig. 6D). Hom mice also showed a trending decrease compared to Het mice (Hom vs. Het, *p* = 0.05) (Fig. 6D). Total sTREM2 levels in Hom mice showed a significant decrease compared to WT mice and a trending decrease compared to Het mice (Hom vs Het, *p* = 0.06) (Fig. 6E). There were no significant differences in the total sTREM2 between Het and WT mice (Fig. 6E). We then observed higher s/fl ratios of TREM2 in Hom and Het mice compared to WT mice (Fig. 6F). No significant differences of s/fl ratios were found between Hom and Het mice. Moreover, in Het mice, TREM2-H157Y showed higher s/fl ratios compared to TREM2-WT (Fig. 6G). Additionally, we measured serum sTREM2 and found an increase in Hom mice compared to WT mice in the presence of brain Aβ, an effect that was independent of brain TREM2 (Fig. 6H).

Similarly, we isolated microglia from WT and Hom mice with amyloid pathology at 8.5 months of age and examined the TREM2 signaling through the detection of pSYK and SYK (Fig. 6I). We observed significant reductions of pSYK levels (Fig. 6J) and pSYK/SYK ratios (Fig. 6L) in the Hom mice compared with the WT mice while there was no difference in the total SYK levels (Fig. 6K), indicating that *Trem2* H157Y suppressed TREM2 signaling in the presence of Aβ.

These results collectively indicate that *Trem2* H157Y facilitates TREM2 shedding, and reduces TREM2 signaling in the brains of 5xFAD mice.

### *Trem2* H157Y downregulates genes related to neuroinflammation in 5xFAD mice

To understand how Trem2 H157Y affects brain transcriptional profiles, we conducted bulk RNA sequencing with brain RNA samples from the non-amyloid-bearing mice at 6 months of age and amyloid-bearing mice at 8.5 months of age.

**Fig. 7:**
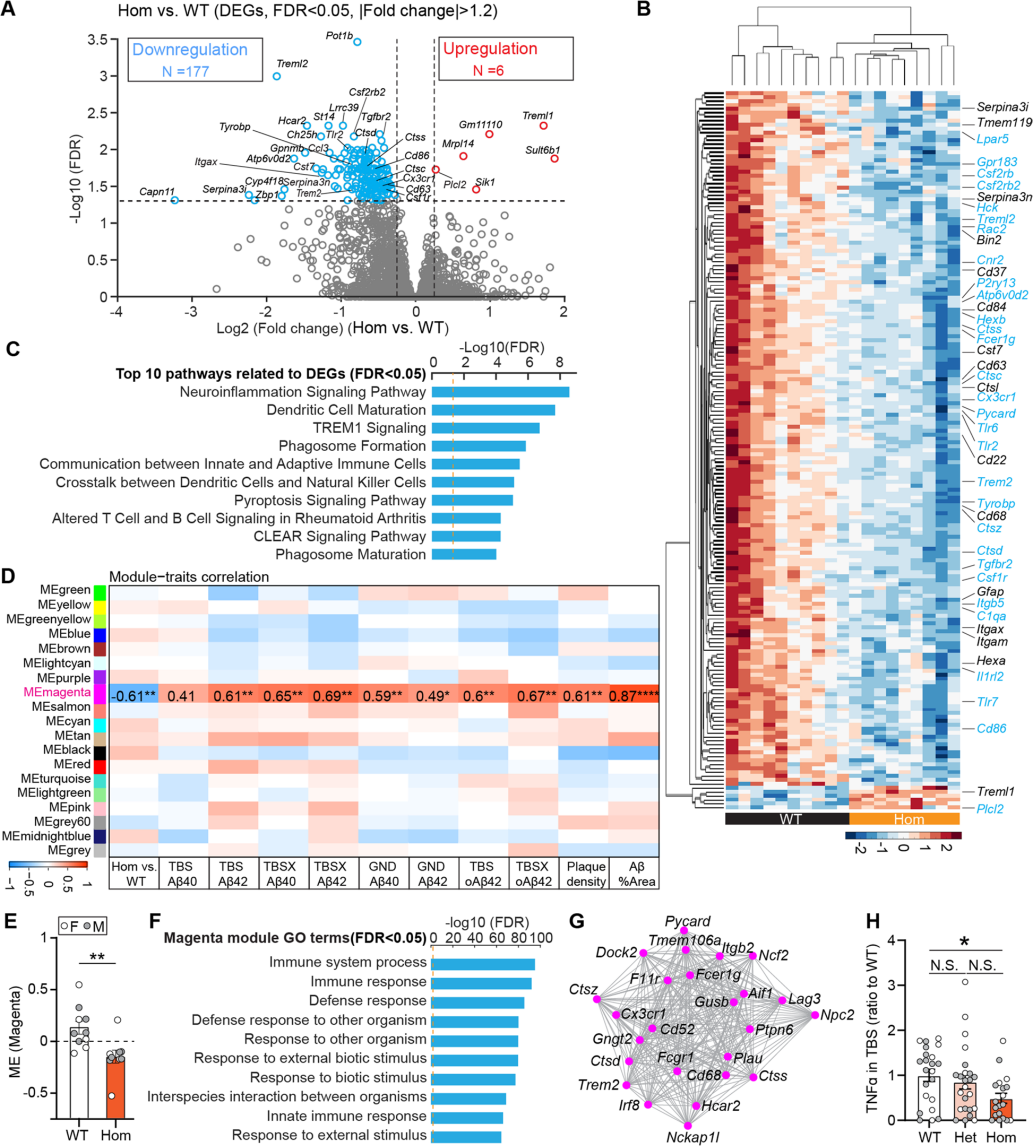
*Trem2* H157Y downregulates genes related to neuroinflammation pathway in 5xFAD mice. Cortical bulk RNA sequencing was conducted with mice at 8.5 months of age (4–5 mice/sex/genotype). ***A***. In the comparison of Hom and WT mice, upregulated or downregulated differentially expressed genes (DEG, Hom vs. WT, |Fold change|> 1.2; FDR< 0.05) were identified and indicated in red or blue in the volcano plot. ***B***. Hierarchical clustering for DEG expression (Hom vs WT) is shown across each sample. DEGs involved in the top 10 pathways (***C***) are shown in blue. ***C***. Top 10 DEG related pathways were identified through Ingenuity Pathway Analysis (IPA). Red dashed line indicates the FDR threshold, 0.05. ***D***. Weighted gene co-expression network analysis (WGCNA) identified a unique magenta module which is downregulated in Hom and correlated with amyloid pathological readouts. ***E***. Magenta module eigengenes (ME) were compared between WT and Hom mice. Data are presented as Mean ± SEM. Wilcoxon rank sum test was used. ***F***. Top 10 gene ontology (GO) terms (FDR < 0.05) are shown for the magenta module. ***G***. Network plot of genes involved in the top 10 GO terms was generated through Cytoscape. ***H***. TNF-α in the TBS lysate was quantified and normalized to WT mice for each genotype. *N* = 19–24 mice per genotype at 8.5 months of age, mixed sex. Data are presented as Mean ± SEM. Kruskal–Wallis tests with uncorrected Dun’s multiple comparisons were used. * *p* < 0.05. **p < 0.01

While there were only three differentially expressed gens (DEGs) identified in the non-amyloid cohort in the comparison of Hom vs. WT mice (Fig. S9A), 183 DEGs emerged responding to amyloid pathology, including 177 downregulated genes and 6 upregulated genes (Fig. 7A). Through hierarchical clustering, we visualized the clear separation of the DEG expression profiles in Hom and WT mice (Fig. 7B). Both disease-associated-microglia (DAM) genes (*Trem2, Tyrobp, Itgax, Cd63, Cst7, Ccl6, Ctsd, Gpnmb, lyz2*, etc.) and microglial homeostatic genes (*Cx3cr1, Tmem119, P2ry12, P2ry13, Hexb, Gpr183*, etc.) [59, 60] were found downregulated in Hom mice compared to WT mice. We validated the changes of the representative genes (*Trem2*, *Tyrobp*, *Tmem119*, *Cx3cr1*, *Cd68*, *C1qa*) in Hom mice compared to WT mice by qPCR (Fig. S9C-G). The downregulation of *Trem2* and *Cd68* was in line with the prior immunostaining results that showed significant reductions of TREM2^+^ (Fig. 4I-K, Fig. 5U-W) and CD68^+^ (Fig. 4E-H, Fig. 5 R-T) areas in the cortex and hippocampus of Hom mice compared to WT mice. On the other hand, several astrocyte abundant genes were also downregulated including *Gfap*, *Serpina3i*, and *Serpina3n* in Hom mice (Fig. 7A-B), which was in line with the significant reductions of GFAP^+^ (Fig. 4P-R, Fig. 5X-Z) areas in the cortex of Hom mice compared to WT mice. Further, the neuroinflammation pathway was shown to be the top DEG-related pathway (Fig. 7C), coinciding with the reduced microglia and astrocyte gliosis in *Trem2* H157Y Hom mice.

We also conducted weighted gene co-expression network analysis (WGCNA) to identify gene modules associated with genotype in the non-amyloid and amyloid cohorts, respectively. Although no significant module was identified related to genotype in the non-amyloid cohort (Fig. S9B), a unique significant magenta module was discovered to be downregulated (Fig. 7D) with significantly lower module eigengenes (MEs) in the Hom mice compared to WT mice (Fig. 7E) in the amyloid cohort. Top gene ontology (GO) of this module pointed to the immune system process (Fig. 7F). Through the abundance examination with the single cell sequencing dataset (http://dropviz.org/) [61], all the 25 genes involved in the top 10 GO terms of the magenta module (Fig. 7G) were found highly expressed in microglia such as *Trem2*, *Cd68*, *Aif1*, *Ctsz*, *Ctsd*, *Cx3cr1*, *Tmem106a* and other 18 genes, suggesting the critical contribution of microglia to the transcriptional changes with the *Trem2* H157Y mutation responding to the amyloid pathology. Further, a significant positive correlation was found between the ME of this immune module and the corresponding amyloid pathological readouts from the same mice (Fig. 7D), suggesting the strong links between reductions of immune responses and amyloid pathology in the Hom mice. Additionally, the magenta module was not preserved in the network built with non-amyloid dataset (Fig. S9H) indicating the specific association of this immune module with amyloid pathology. In line with the immune process downregulation, we observed significant reductions of inflammatory cytokine, TNFα in the TBS lysates of Hom mice compared to WT mice while no significant differences between Het mice and WT or Hom mice were found (Fig. 7H).

Taken together, *Trem2* H157Y downregulated microglial genes involved in the neuroinflammation signaling pathway in Hom mice.

## Discussion

In this study, we provided in vivo evidence that *Trem2* H157Y mutation promotes TREM2 shedding in our novel *Trem2* H157Y knock-in mouse models. Moreover, we found *Trem2* H157Y enhances synaptic plasticity, facilitates Aβ clearance, reduces amyloid burden and gliosis, and downregulates neuroinflammatory pathways.

Consistent with previous in vitro findings [23, 24], we observed significantly higher sTREM2 level in cortical TBS lysates, conditioned medium of primary microglia, and peripheral serum from mice with the *Trem2* H157Y mutation. We did not observe significant changes in the membrane associated flTREM2 levels and TREM2 signaling. Since N-terminal TREM2 ELISA does not distinguish mature and immature flTREM2, we cannot conclude that *Trem2* H157Y specifically reduces mature TREM2 in our mouse model as described in the in vitro studies [23, 24]. On the other hand, in the presence of Aβ, we observed significant reductions of sTREM2, flTREM2, cortical and hippocampal TREM2^+^ areas, as well as TREM2 signaling in Hom mice compared to WT mice at 8.5 months of age. Despite the reduction of total TREM2 levels, TREM2-H157Y displays higher s/fl ratios compared to TREM2-WT, suggesting that *Trem2* H157Y facilitates TREM2 shedding in the 5xFAD mice. In addition, similar to the non-amyloid cohort, we observed elevated serum sTREM2 in Hom mice compared to WT mice. Collectively, our in vivo studies suggest that *Trem2* H157Y promotes TREM2 shedding in the absence or presence of Aβ.

Moreover, we observed enhanced presynaptic facilitation and synaptic plasticity in Hom mice and a trending improvement of spatial working memory in Het mice, along with the increased sTREM2 from microglia. It has been shown that sTREM2 was colocalized with neurons [21] which implicates the involvement of sTREM2 in neuronal function. Thus, effects of *Trem2* H157Y on synaptic functions in our mouse models may be mediated, but not restricted to sTREM2, through a microglia non-cell autonomous mechanism. To address our hypothesis and elucidate the exact roles of TREM2 and sTREM2 in regulating neuronal activity, more comprehensive studies are needed in future studies.

We detected ~ 0.4 and ~ 0.6 ng sTREM2/mg TBS proteins in the TBS lysates from WT and Hom mice, i.e., ~ 0.4 and ~ 0.6 fmol sTREM2/mg brain tissue, respectively. These amounts are at the same order of magnitude as the mass spectrometry measurements in the amyloid mice (Fig. 6E, 8–15 fmol/mg brain tissue), indicating the reliability of the TREM2 ELISA method. Despite the significantly increased TREM2 shedding in the Hom mice, we did not observe changes in microglial morphology and density in the non-amyloid *Trem2* H157Y knock-in mice. We also did not observe significant cortical transcriptional profile changes in Hom mice compared to WT mice. However, a study from Zhong et al., reported that the application of exogenous sTREM2 increases microglia survival, stimulate microglia to a reactive status, and induce proinflammatory cytokine production [62]. Specifically, they locally injected 3 µg sTREM2 (~ 0.1 nmol) to the hippocampus, i.e., ~ 3.3 pmol sTREM2/mg tissue given that the weight of hippocampus is ~ 30 mg. This exogenous sTREM2 amount is much more than the endogenous measurements in our mouse models. Therefore, we speculate that the low amount of endogenous brain sTREM2 in our mice may not be sufficient to result in significant microglial changes in morphology and proinflammatory responses as found in that study.

At the transcriptional level, we observed a suppression of DAM gene expression, downregulation of a microglia-related immune module, and reduction of inflammatory cytokines in Hom mice. This hypoimmune status of the brain may result from the general suppression of microgliosis responding to the reduced amyloid burden in *Trem2* H157Y homozygous mice. These results are in line with the work from Dhandapani, R., et al., which reported that reduced TREM2 shedding advances the DAM signatures of microglia, promotes immune response, and aggregates Aβ pathology [63]. Our study and their work together suggest the critical role of sTREM2 in microglial response to amyloid pathology. Future dissection of microglial transcriptional profile at the single cell level in our mouse models will lead to a better understanding of sTREM2 in regulating microglia status and Aβ pathology.

The mechanism by which *Trem2* H157Y facilitates Aβ clearance and lowers amyloid burden is not well understood. However, we speculate that this might link to the interaction between sTREM2 and Aβ oligomer. It has been shown that the Aβ oligomer can bind to TREM2 or sTREM2 [12–14, 64]. Also, Aβ oligomers stimulate sTREM2 production in a dose-dependent manner in vitro and sTREM2 in return inhibits Aβ aggregation [14] likely through suppressing secondary nucleation of Aβ fibrillization [65]. Soluble TREM2 also enhances cellular uptake of fibrillar Aβ in H4 and HMC cells [65]. All these findings suggest that sTREM2 could facilitate Aβ diffusion and clearance in vivo. On the other hand, studies have shown that elevating sTREM2 through exogenous administration or AAV-mediated overexpression significantly reduces amyloid burden [66] even though the amount of sTREM2 they applied is greater than the endogenous level measured in our mouse model. Depleting microglia abolishes the rescuing effect of sTREM2, suggesting that sTREM2 may reduce amyloid load through microglia signaling [66]. Thus, in our mouse models, increased TREM2 shedding in *Trem2* H157Y homozygous mice may accelerate Aβ clearance and promote microglial uptake of fibrillar Aβ, leading to the overall decrease of amyloid burden and related microgliosis.

On the other hand, studies on *Trem2* p.R47H reveal a loss of TREM2 function in ligand binding, signaling, and microglial responses to pathological cues [6, 21], which inspired the development of TREM2 activating antibodies to alleviate AD pathology. TREM2 antibody administration in amyloid mouse models has been found to boost microglial responses to Aβ, reduce amyloid load, toxicity, and behavioral impairments [67–70]. TREM2 activating antibodies stabilize the membrane associated TREM2 and related signaling. Accordingly, the levels of sTREM2 in serum and CSF decrease in an antibody-dose dependent manner in mice and humans [68–70]. These findings emphasize the critical role of membrane bound TREM2 in cell-autonomous microglia activation and phagocytosis to affect amyloid pathology. Using *Trem2* H157Y knock-in mouse models, our data alternatively suggests non-cell autonomous benefits of sTREM2 on synaptic function and Aβ clearance, encouraging a consideration of increasing sTREM2 as a potential therapeutic strategy to treat AD. Combination therapy by activating TREM2 signaling and elevating sTREM2 level should also be considered.

## Conclusion

In summary, our study confirmed the increased shedding of TREM2-H157Y in vivo and defined beneficial effects of *Trem2* H157Y in brain function and in reducing amyloid pathology. However, these findings conflict with the genetic studies showing the increased AD risk associated with *TREM2* p.H157Y. Considering that no animal model fully mimics the AD related pathologies and 5xFAD mice merely develop amyloid pathology which recapitulates the very early stage of AD [71], our current data cannot address how *TREM2* p.H157Y affects late-stage AD pathologies including tauopathy and neurodegeneration. Moreover, in AD patients, sTREM2 mediates earliest amyloid-associated phosphorylated tau increases [72] and is associated with tau related-neurodegeneration but not with Aβ pathology [73]. Thus, more investigations are necessary to further elucidate the effects of *TREM2* H157Y mutation on other AD pathogenic events, in particular the tau pathology and related neurodegeneration.

## Supplementary Information

**Additional file 1: Figure S1.** Analysis of potential off target effects in the *Trem2* H157Y knock-in mice. A. Top three putative off targets (*A*) with Cutting Frequency Determination (CFD) Score ranging from 0.28 to 0.44 were identified and sequenced with primers accordingly. *B*. Single peaks were seen at the putative sites (highlighted in red, arrowhead), while two signals were seen at the Trem2 H157Y target site (highlighted in red, arrowhead). Orange arrows indicate the putative region and direction recognized by gRNA.

**Additional file 2: Figure S2.***Trem2* H157Y does not affect microglia density and morphology. *A*. Representative images of IBA1 staining are shown for WT, Het, and Hom mice at 6 months of age. Scale, 400 µm. *B-C*. Cortical microglia (MG) number (*B*) and cell body size (*C*) are quantified in Image J for each genotype at 6 months of age. *N* =11-14 mice per genotype, mixed sex. *D-F*. Representative confocal images (*D*) of IBA1 staining were processed (*E*) and skeletonized (*F*) in image J for each genotype at 6 months of age. Scale bar for D and E, 50 µm; Scale bar for F 10 µm. *G-I*. The branch number (G), junction number (H), and total branch length per microglia (MG) (*I*) were assessed for each genotype at 6 months of age. *N* = 9-10 mice per genotype, mixed sex. Data are presented as Mean±SEM. Kruskal-Wallis tests with uncorrected Dun’s multiple comparisons were used were used in *B-C, G-I. N.S*., not significant.

**Additional file 3: Figure S3.** *Trem2* H157Y does not affect synaptic integrity, anxiety, working memory and associative memory. *A*. Synaptophysin, PSD95, and GLUR2 were detected in TBSX lysates. *B-D*. Synaptophysin (*B*), PSD95 (*C*), and GLUR2 (*D*), were quantified and normalized to WT. *N* = 9-10 mice per genotype at 6 months of age, mixed sex. E. Open field analysis (OFA) was conducted to examine the anxiety of mice with different genotypes at 6 months of age. *N* =37-40 mice per genotype, mixed sex. F. Y-maze spontaneous alteration test was conducted to examine the working memory of mice with different genotypes at 6 months of age. *N* =23-26 mice per genotype, mixed sex. *G*. Contextual fear conditioning test (CFC) was conducted to examine the associative memory of mice with different genotypes at 6 months of age. *N* =37-40 mice per genotype, mixed sex. *H*. Cued fear conditioning test (CFC) was conducted to examine the associative memory of mice with different genotypes at 6 months of age. *N* =37-40 mice per genotype, mixed sex. B-D, E-H. Data are presented as Mean±SEM. Kruskal-Wallis tests with uncorrected Dun’s multiple comparisons were used. N.S., not significant.

**Additional file 4: Figure S4.** *Trem2* H157Y doesn’t affect amyloid pathology in 5xFAD mice at 4 months of age. *A-B*. Representative images of pan-amyloid (MOAB2, *A*) and fibrillar amyloid (X34, *B*) staining are shown at 4 months of age. Scale, 400 µm. *C-H*. Cortical (*C-E*) and hippocampal (*F-H*) amyloid plaque area coverages (*C, F*), densities (*D, G*) and sizes (*E, H*) are quantified and normalized to WT for each genotype. I-N. Cortical (*I-K*) and hippocampal (*L-N*) fibrillar amyloid plaque area coverages (*I, L*), densities (J, M) and sizes (K, N) are quantified and normalized to WT. O-T. Aβ40 (O, Q, S) and Aβ42 (*P*, *R*, *T*) were quantified by ELISA and normalized to WT in cortical TBS (*O, P*), TBSX (*Q, R*) and GND (*S, T*) for each genotype. *C-T*. *N* = 12-18 mice per genotype at 4 months of age, Data are presented as Mean±SEM. Kruskal-Wallis tests with uncorrected Dun’s multiple comparisons were used. N.S., not significant.

**Additional file 5: Figure S5.** *Trem2* H157Y does not affect microgliosis and astrogliosis in 5xFAD mice at 4 months of age. A. Representative images of IBA1 staining are shown for each genotype at 4 months of age. Scale, 400 µm. *B-C*, Cortical (*B*) and hippocampal (*C*) IBA1^+^ area coverages are quantified and normalized to WT. *D*. Representative images of GFAP staining are shown for each genotype at 4 months of age. Scale, 400 µm. *E-F*. Cortical (*E*) and hippocampal (*F*) GFAP^+^ area coverages were quantified and normalized to WT for each genotype. *B-C, E-F*. *N* = 12-18 mice/genotype at 4 months of age. Data are presented as Mean±SEM. Kruskal-Wallis tests with uncorrected Dun’s multiple comparisons were used. N.S., not significant.

**Additional file 6: Figure S6.** Effects of *Trem2* H157Y mutation on Aβ levels, neuronal toxicity, and APP processing at 8.5 months of age. *A-D*. Aβ40 (*A, C*) and Aβ42 (*B, D*) were quantified by ELISA, and normalized to WT in cortical TBS (*A, B*), and TBSX (*C, D*) for each genotype. *E*. Representative images of fibrillar amyloid (X34) staining are shown for each genotype at 8.5 months of age. Scale, 400 µm. *F-K*. Cortical (*F-H*) and hippocampal (*I-K*) fibrillar amyloid plaque area coverages (*F, I*), densities (*G, J*) and sizes (*H, K*) are quantified and normalized to WT. *L*. Representative images of APP (C-terminal APP antibody) staining are shown for each genotype at 8.5 months of age. Scale, 400 µm. *M-N*. Cortical (*B*) and hippocampal (*C*) APP^+^ area coverages were quantified and normalized to WT. *O*. Representative gel images are shown for synaptophysin and PSD95, in TBSX lysates with β-Actin stain for normalization. *P-Q*. PSD95 (*P*) and Synaptophysin (*Q*), were quantified and normalized to WT. *R-S*. Soluble APPa (sAPPa, *R*), Soluble APPβ (sAPPβ, *S*) were examined by ELISA, quantified, and normalized to WT in TBS lysates for each genotype. T. CTFβ was examined by ELISA, quantified, and normalized to WT in TBSX lysates for each genotype. A-T. *N* = 19-24 mice per genotype at 8.5 months of age, mixed sex. Data are presented as Mean±SEM. Kruskal-Wallis tests with uncorrected Dun’s multiple comparisons were used. N.S., not significant. * *p*<0.05.

**Additional file 7: Figure S7.** Effects of *Trem2* H157Y mutation on Aβ levels and neuronal dystrophy in founder 2# linage 5xFAD mice at plateau stage of amyloid development. A-D. Aβ40 (A, C) and Aβ42 (B, D) were quantified by ELISA, and normalized to WT in cortical TBS (A, B), and TBSX (C, D) for each genotype. E. Representative images of fibrillar amyloid (X34) staining are shown for each genotype at 8.5 months of age. Scale, 400 µm. F-K. Cortical (F-H) and hippocampal (I-K) fibrillar amyloid plaque area coverages (F, I), densities (G, J) and sizes (H, K) are quantified and normalized to WT for each genotype. L. Representative images of APP (C-terminal APP antibody) staining are shown for each genotype at 8.5 months of age. Scale, 400 µm. M-N. Cortical (M) and hippocampal (N) APP^+^ area coverages are quantified and normalized to WT for each genotype. A-N. *N* = 8-13 mice per genotype at 8.5 months of age, mixed sex. Data are presented as Mean±SEM. Kruskal-Wallis tests with uncorrected Dun’s multiple comparisons were used. N.S., not significant. * *p*<0.05.

**Additional file 8: Figure S8.** Identification and measurements of full-length, soluble TREM2-WT and TREM2-H157Y in mouse brain through mass spectrometry. A. Workflow of the TREM2-targeted mass spectrometry is illustrated. B. TREM2 was detected through an N-terminal antibody (5F4) in the input, supernatant and eluent samples from TREM2 immunoprecipitation through a biotinylated N-terminal antibody (BAF1729) in brain lysates of 5xFAD^+/-^ and TREM2^-/-^ mice. C. Signature peptide sequences for flTREM2-WT, sTREM2-WT, flTREM2-H157Y and sTREM2-H157Y are shown. D-G, Annotated MS/MS spectra of four unique peptides from flTREM2-WT (D), sTREM2-WT (E), flTREM2-H157Y (F) and sTREM2-H157Y (G), respectively. H-K. Measurements of flTREM2-WT (H), sTREM2-WT (I), flTREM2-H157Y (J) and sTREM2-H157Y (***K***) in brain samples from each genotype of 5xFAD mice. N = 3 mice/sex/genotype at 8.5 months of age. Data are presented as Mean±SEM. Ordinary one-way ANOVA with uncorrected Fisher's LSD multiple comparisons were used. N.S., not significant. *p < 0.01. **p < 0.01. ***p < 0.001. ****p < 0.0001.

**Additional file 9: Figure S9.** Effects of *Trem2* H157Y on the transcriptome profiles. A. Three DEGs (Hom vs. WT, |Fold change|>1.2; FDR<0.05) were identified in the non-amyloid mice. N=5 mice/sex/genotype at 6 months of age. B. No significant modules were identified related to genotype in the non-amyloid cohort. C-G. DEGs, *Trem2*, *Tyrobp*, *Tmem119*, *Cx3cr1*, and *C1q* were validated through qPCR in 5xFAD mice. N = 19-24 mice per genotype at 8.5 months of age, mixed sex. Data are presented as Mean±SEM. Kruskal-Wallis tests with uncorrected Dun’s multiple comparisons were used. N.S., not significant. * *p*<0.05. ** *p*<0.01. *** *p*<0.001. **** *p*<0.0001. H. The magenta module identified in amyloid mice was not preserved in the non-amyloid network revealed by a low preservation Zsummary value (<2).

**Additional file 10: Figure S10.** A working model is illustrated demonstrating the hypothesis that *Trem2* H157Y reduces amyloid load through facilitating Aβ clearance mediated by sTREM2 which binds to Aβ oligomers and fibrils, and inhibit plaque formation. The reduced amyloid load leads to downregulated immune responses of microglia with less cytokine released.

## Data Availability

The source data for each figure are available from the corresponding author on reasonable request. Bulk RNA sequencing data are available in NCBI’s Gene Expression Omnibus with the accession number GSE212618.

## Abbreviations

AD: Alzheimer’s Disease
Aβ: Amyloid β
TREM2: Triggering Receptor expressed on myeloid cells 2
flTREM2: Full-length TREM2
CRISPR: Clustered regularly interspaced short palindromic repeats
Cas9: CRISPR-associated protein 9
gRNA: guide RNA
GND: Guanidine
PFA: paraformaldehyde
AP: Anteroposterior
RT: Room temperature
WT: Wild type
Het: Heterozygous
Hom: Homozygous
ISF: Interstitial fluid
aCSF: Artificial cerebrospinal fluid
PRM: Parallel reaction monitoring
RIN: RNA integrity numbers
qPCR: Quantitative PCR.
sTREM2: Soluble TREM2
MG: Microglia
IBA1: Ionized calcium-binding adapter molecule 1
LAMP1: Lysosome-associated membrane protein
LTP: Long term potential
PPF: Paired-pulse facilitation
DEG: Differentially expressed genes
FC: Fold change
WGCNA: Weighted gene co-expression network analysis
DAM: Disease-associated-microglia
LDL: low-density lipoprotein
HDL: High-density lipoprotein
FDR: false discovery rate
