# Correction: *Trem2* H157Y increases soluble TREM2 production and reduces amyloid pathology

**DOI:** 10.1186/s13024-023-00603-w

**Published:** 2023-04-28

**Authors:** Wenhui Qiao, Yixing Chen, Jun Zhong, Benjamin J. Madden, Cristine M. Charlesworth, Yuka A. Martens, Chia-Chen Liu, Joshua Knight, Tadafumi C. Ikezu, Aishe Kurti, Yiyang Zhu, Axel Meneses, Cassandra L. Rosenberg, Lindsey A. Kuchenbecker, Lucy K. Vanmaele, Fuyao Li, Kai Chen, Francis Shue, Maxwell V. Dacquel, John Fryer, Akhilesh Pandey, Na Zhao, Guojun Bu

**Affiliations:** 1 Department of Neuroscience, Mayo Clinic, Jacksonville, FL 32224 USA; 2 Department of Laboratory Medicine and Pathology, Mayo Clinic, Rochester, MN 55905 USA; 3 Medical Genome Facility, Proteomics Core, Mayo Clinic, Rochester, MN USA; 4 Department of Neuroscience, Mayo Clinic, Scottsdale, AZ 85259 USA; 5 Center for Individualized Medicine, Mayo Clinic, Rochester, MN USA; 6 Manipal Academy of Higher Education (MAHE), Manipal, Karnataka 576104 India; 7 SciNeuro Pharmaceuticals, Rockville, MD 20805 USA

**Correction: Molecular Neurodegeneration 18, 8 (2023)**

**https://doi.org/10.1186/s13024-023-00599-3**

The original article [1] contained numerous typos and errors which have since been amended.
