# Supplementary figures and images for: Trem2 H157Y increases soluble TREM2 production and reduces amyloid pathology
Source: Mol Neurodegener. 2023 Jan 31;18:8. doi: 10.1186/s13024-023-00599-3 (PMC9890893; doi:10.1186/s13024-023-00599-3)

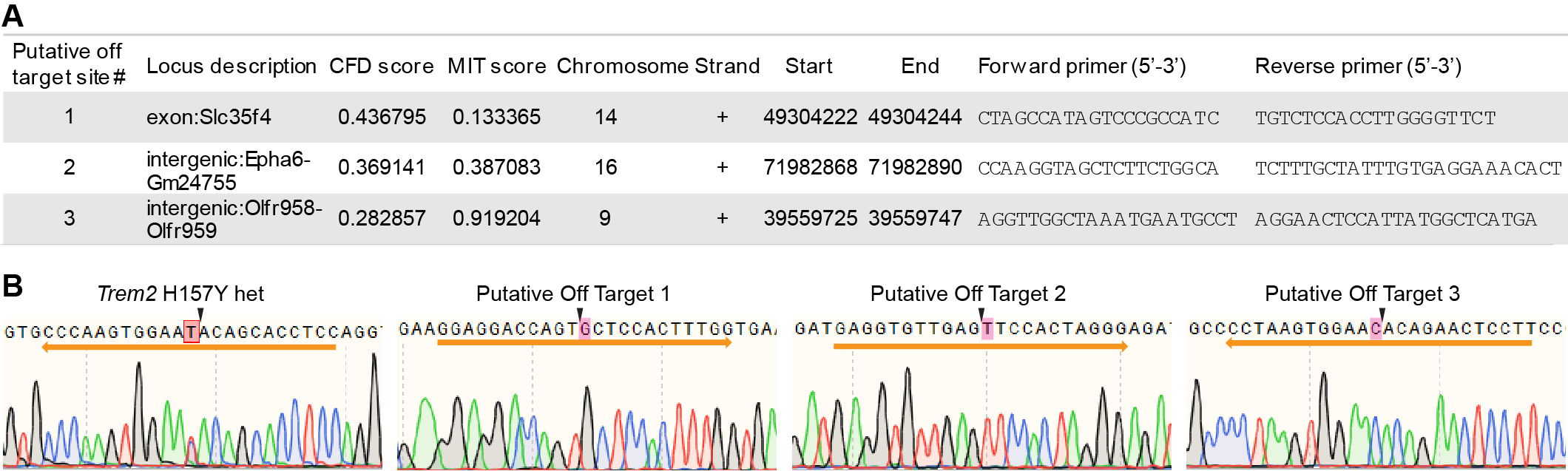

Supplement: Supplementary file 1 — Additional file 1: Figure S1. Analysis of potential off target effects in the Trem2 H157Y knock-in mice. A. Top three putative off targets (A) with Cutting Frequency Determination (CFD) Score ranging from 0.28 to 0.44 were identified and sequenced with primers accordingly. B. Single peaks were seen at the putative sites (highlighted in red, arrowhead), while two signals were seen at the Trem2 H157Y target site (highlighted in red, arrowhead). Orange arrows indicate the putative region and direction recognized by gRNA. [file 13024_2023_599_MOESM1_ESM.png]

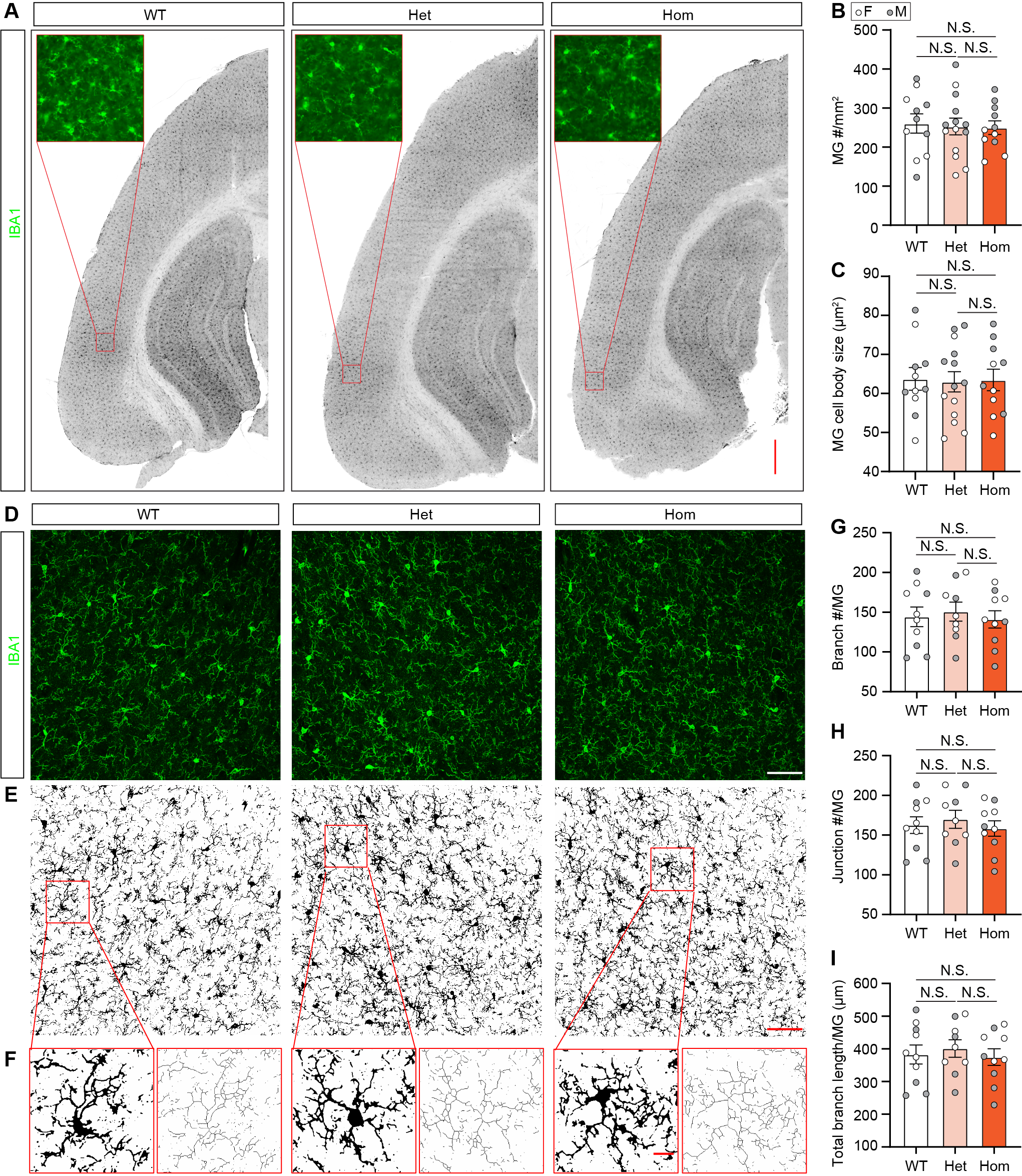

Supplement: Supplementary file 2 — Additional file 2: Figure S2.Trem2 H157Y does not affect microglia density and morphology. A. Representative images of IBA1 staining are shown for WT, Het, and Hom mice at 6 months of age. Scale, 400 µm. B-C. Cortical microglia (MG) number (B) and cell body size (C) are quantified in Image J for each genotype at 6 months of age. N =11-14 mice per genotype, mixed sex. D-F. Representative confocal images (D) of IBA1 staining were processed (E) and skeletonized (F) in image J for each genotype at 6 months of age. Scale bar for D and E, 50 µm; Scale bar for F 10 µm. G-I. The branch number (G), junction number (H), and total branch length per microglia (MG) (I) were assessed for each genotype at 6 months of age. N = 9-10 mice per genotype, mixed sex. Data are presented as Mean±SEM. Kruskal-Wallis tests with uncorrected Dun’s multiple comparisons were used were used in B-C, G-I. N.S., not significant. [file 13024_2023_599_MOESM2_ESM.png]

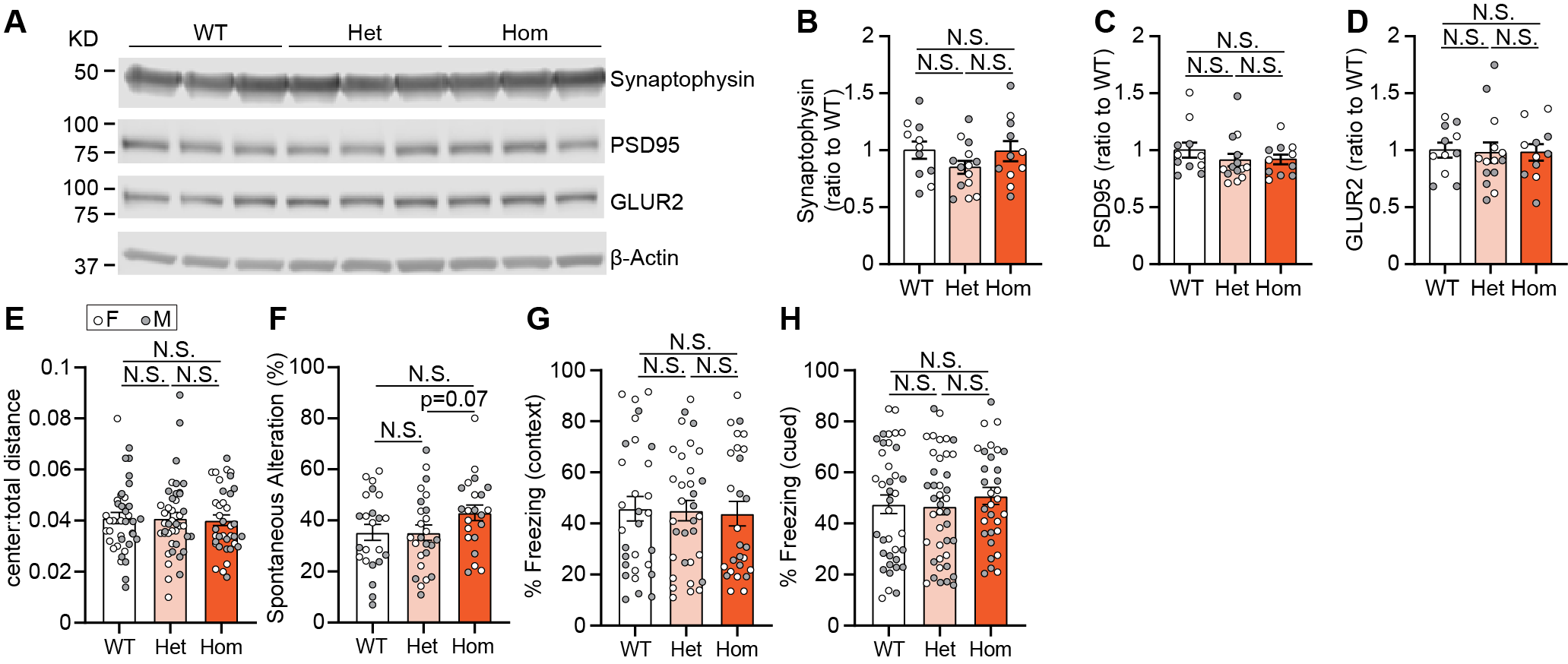

Supplement: Supplementary file 3 — Additional file 3: Figure S3. Trem2 H157Y does not affect synaptic integrity, anxiety, working memory and associative memory. A. Synaptophysin, PSD95, and GLUR2 were detected in TBSX lysates. B-D. Synaptophysin (B), PSD95 (C), and GLUR2 (D), were quantified and normalized to WT. N = 9-10 mice per genotype at 6 months of age, mixed sex. E. Open field analysis (OFA) was conducted to examine the anxiety of mice with different genotypes at 6 months of age. N =37-40 mice per genotype, mixed sex. F. Y-maze spontaneous alteration test was conducted to examine the working memory of mice with different genotypes at 6 months of age. N =23-26 mice per genotype, mixed sex. G. Contextual fear conditioning test (CFC) was conducted to examine the associative memory of mice with different genotypes at 6 months of age. N =37-40 mice per genotype, mixed sex. H. Cued fear conditioning test (CFC) was conducted to examine the associative memory of mice with different genotypes at 6 months of age. N =37-40 mice per genotype, mixed sex. B-D, E-H. Data are presented as Mean±SEM. Kruskal-Wallis tests with uncorrected Dun’s multiple comparisons were used. N.S., not significant. [file 13024_2023_599_MOESM3_ESM.png]

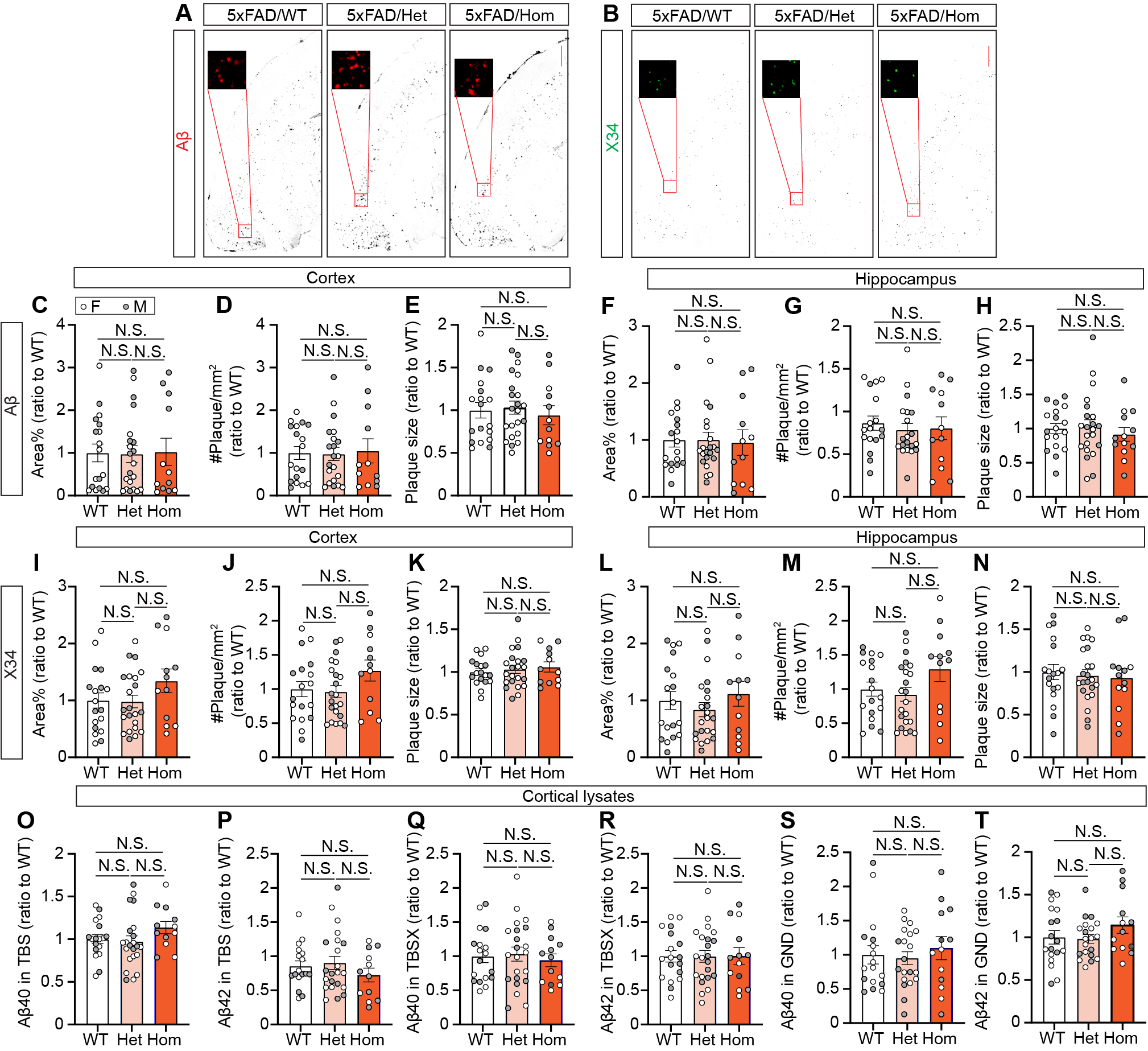

Supplement: Supplementary file 4 — Additional file 4: Figure S4. Trem2 H157Y doesn’t affect amyloid pathology in 5xFAD mice at 4 months of age. A-B. Representative images of pan-amyloid (MOAB2, A) and fibrillar amyloid (X34, B) staining are shown at 4 months of age. Scale, 400 µm. C-H. Cortical (C-E) and hippocampal (F-H) amyloid plaque area coverages (C, F), densities (D, G) and sizes (E, H) are quantified and normalized to WT for each genotype. I-N. Cortical (I-K) and hippocampal (L-N) fibrillar amyloid plaque area coverages (I, L), densities (J, M) and sizes (K, N) are quantified and normalized to WT. O-T. Aβ40 (O, Q, S) and Aβ42 (P, R, T) were quantified by ELISA and normalized to WT in cortical TBS (O, P), TBSX (Q, R) and GND (S, T) for each genotype. C-T. N = 12-18 mice per genotype at 4 months of age, Data are presented as Mean±SEM. Kruskal-Wallis tests with uncorrected Dun’s multiple comparisons were used. N.S., not significant. [file 13024_2023_599_MOESM4_ESM.png]

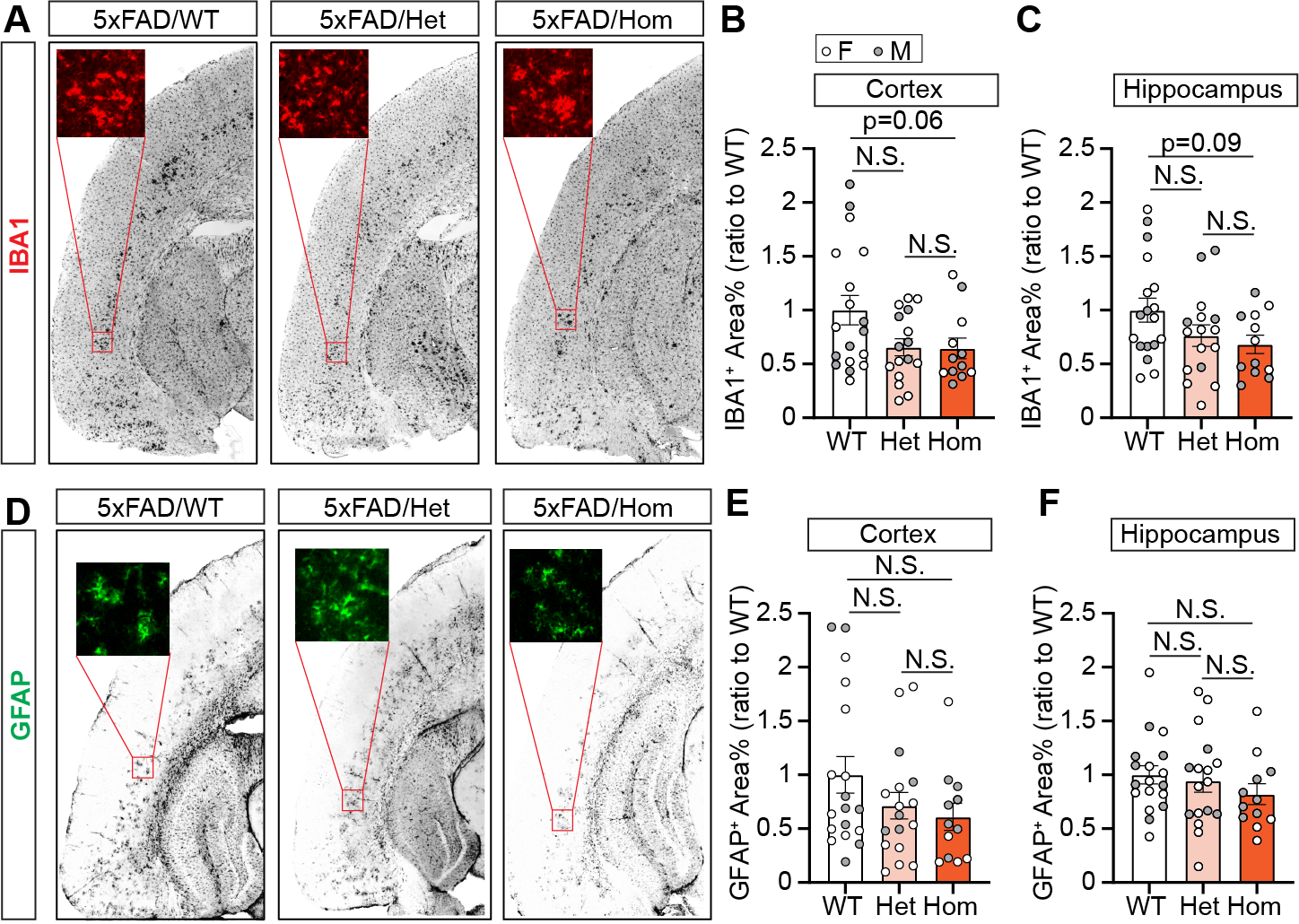

Supplement: Supplementary file 5 — Additional file 5: Figure S5. Trem2 H157Y does not affect microgliosis and astrogliosis in 5xFAD mice at 4 months of age. A. Representative images of IBA1 staining are shown for each genotype at 4 months of age. Scale, 400 µm. B-C, Cortical (B) and hippocampal (C) IBA1+ area coverages are quantified and normalized to WT. D. Representative images of GFAP staining are shown for each genotype at 4 months of age. Scale, 400 µm. E-F. Cortical (E) and hippocampal (F) GFAP+ area coverages were quantified and normalized to WT for each genotype. B-C, E-F. N = 12-18 mice/genotype at 4 months of age. Data are presented as Mean±SEM. Kruskal-Wallis tests with uncorrected Dun’s multiple comparisons were used. N.S., not significant. [file 13024_2023_599_MOESM5_ESM.png]

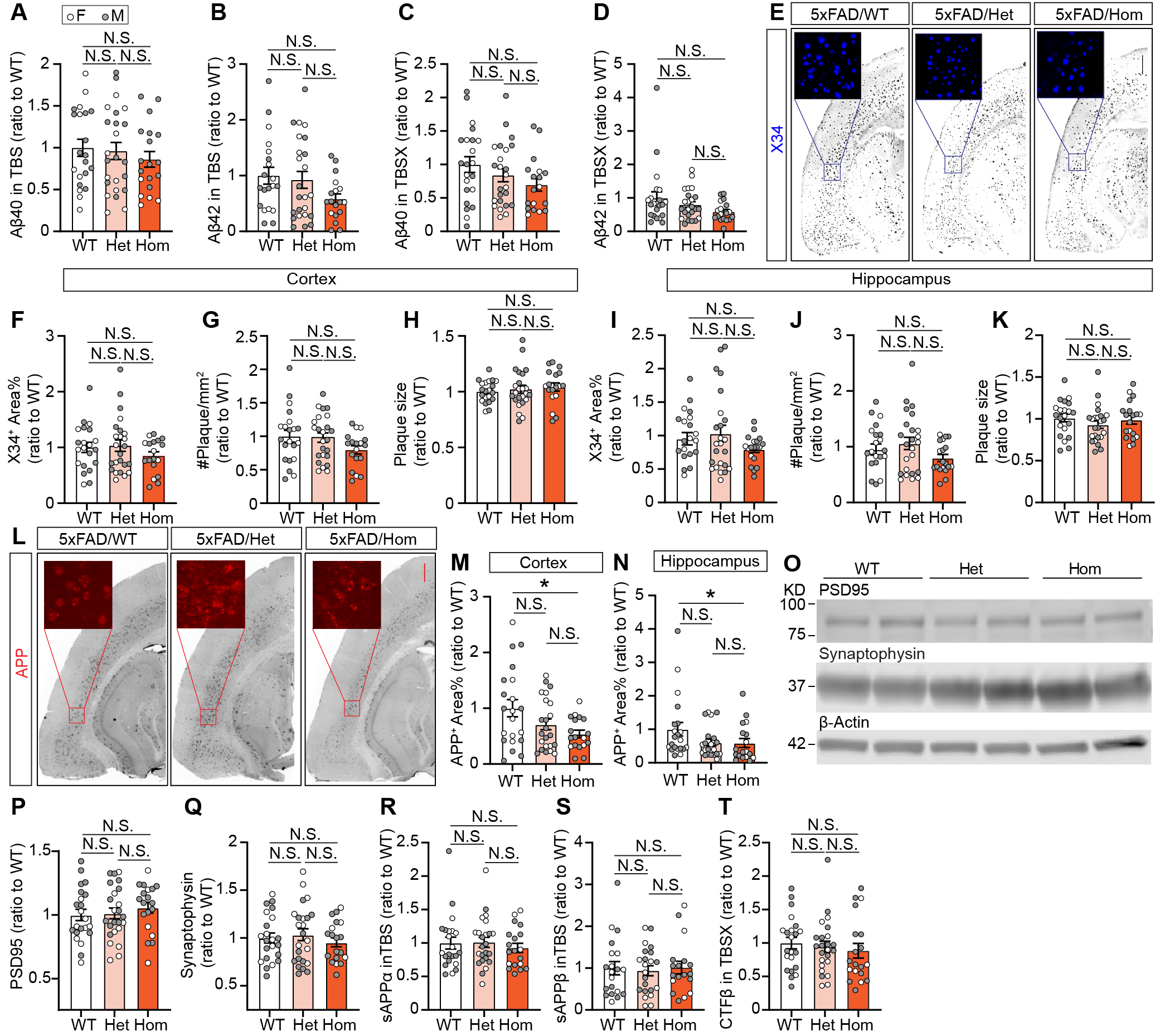

Supplement: Supplementary file 6 — Additional file 6: Figure S6. Effects of Trem2 H157Y mutation on Aβ levels, neuronal toxicity, and APP processing at 8.5 months of age. A-D. Aβ40 (A, C) and Aβ42 (B, D) were quantified by ELISA, and normalized to WT in cortical TBS (A, B), and TBSX (C, D) for each genotype. E. Representative images of fibrillar amyloid (X34) staining are shown for each genotype at 8.5 months of age. Scale, 400 µm. F-K. Cortical (F-H) and hippocampal (I-K) fibrillar amyloid plaque area coverages (F, I), densities (G, J) and sizes (H, K) are quantified and normalized to WT. L. Representative images of APP (C-terminal APP antibody) staining are shown for each genotype at 8.5 months of age. Scale, 400 µm. M-N. Cortical (B) and hippocampal (C) APP+ area coverages were quantified and normalized to WT. O. Representative gel images are shown for synaptophysin and PSD95, in TBSX lysates with β-Actin stain for normalization. P-Q. PSD95 (P) and Synaptophysin (Q), were quantified and normalized to WT. R-S. Soluble APPa (sAPPa, R), Soluble APPβ (sAPPβ, S) were examined by ELISA, quantified, and normalized to WT in TBS lysates for each genotype. T. CTFβ was examined by ELISA, quantified, and normalized to WT in TBSX lysates for each genotype. A-T. N = 19-24 mice per genotype at 8.5 months of age, mixed sex. Data are presented as Mean±SEM. Kruskal-Wallis tests with uncorrected Dun’s multiple comparisons were used. N.S., not significant. * p<0.05. [file 13024_2023_599_MOESM6_ESM.png]

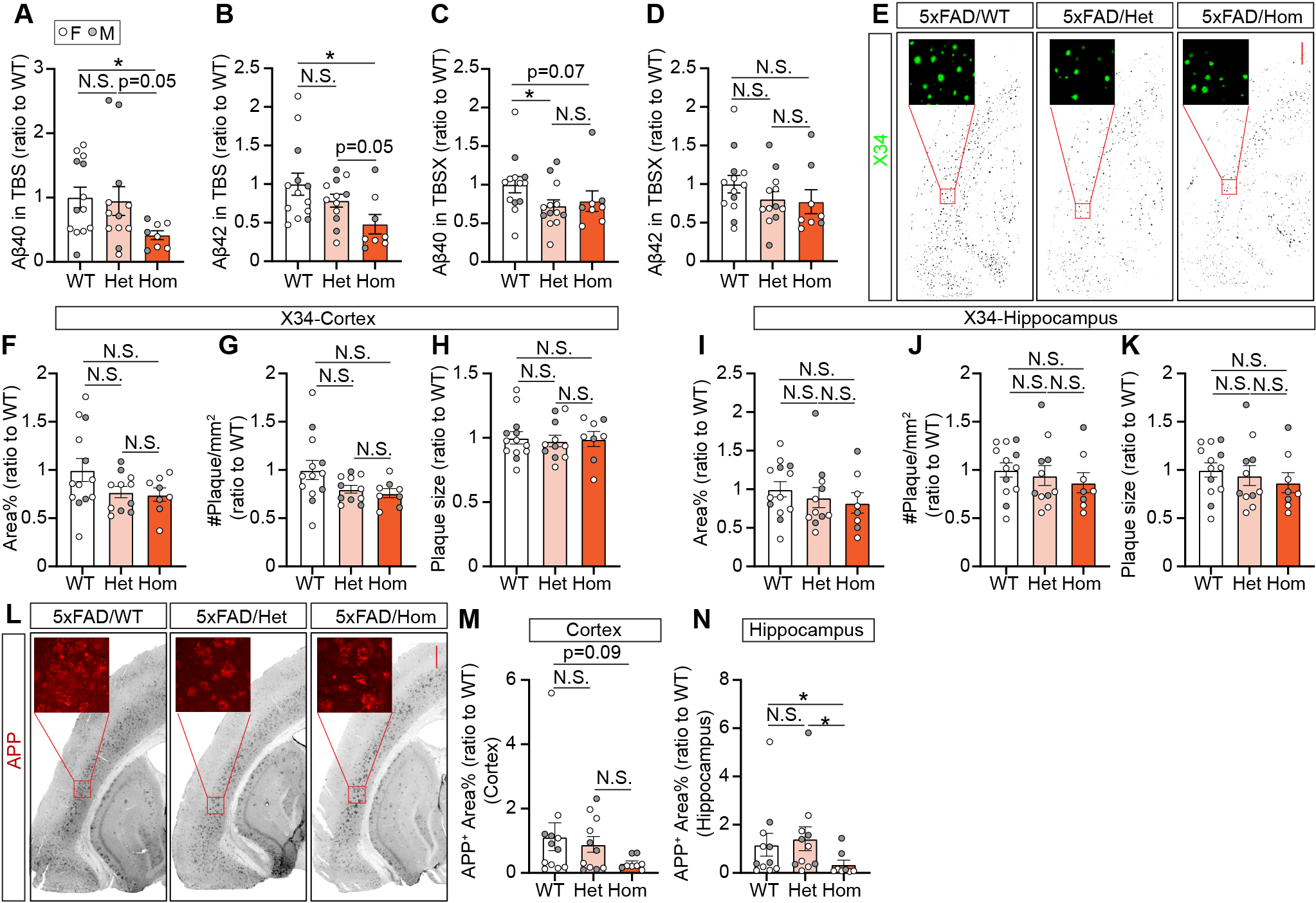

Supplement: Supplementary file 7 — Additional file 7: Figure S7. Effects of Trem2 H157Y mutation on Aβ levels and neuronal dystrophy in founder 2# linage 5xFAD mice at plateau stage of amyloid development. A-D. Aβ40 (A, C) and Aβ42 (B, D) were quantified by ELISA, and normalized to WT in cortical TBS (A, B), and TBSX (C, D) for each genotype. E. Representative images of fibrillar amyloid (X34) staining are shown for each genotype at 8.5 months of age. Scale, 400 µm. F-K. Cortical (F-H) and hippocampal (I-K) fibrillar amyloid plaque area coverages (F, I), densities (G, J) and sizes (H, K) are quantified and normalized to WT for each genotype. L. Representative images of APP (C-terminal APP antibody) staining are shown for each genotype at 8.5 months of age. Scale, 400 µm. M-N. Cortical (M) and hippocampal (N) APP+ area coverages are quantified and normalized to WT for each genotype. A-N. N = 8-13 mice per genotype at 8.5 months of age, mixed sex. Data are presented as Mean±SEM. Kruskal-Wallis tests with uncorrected Dun’s multiple comparisons were used. N.S., not significant. * p<0.05. [file 13024_2023_599_MOESM7_ESM.png]

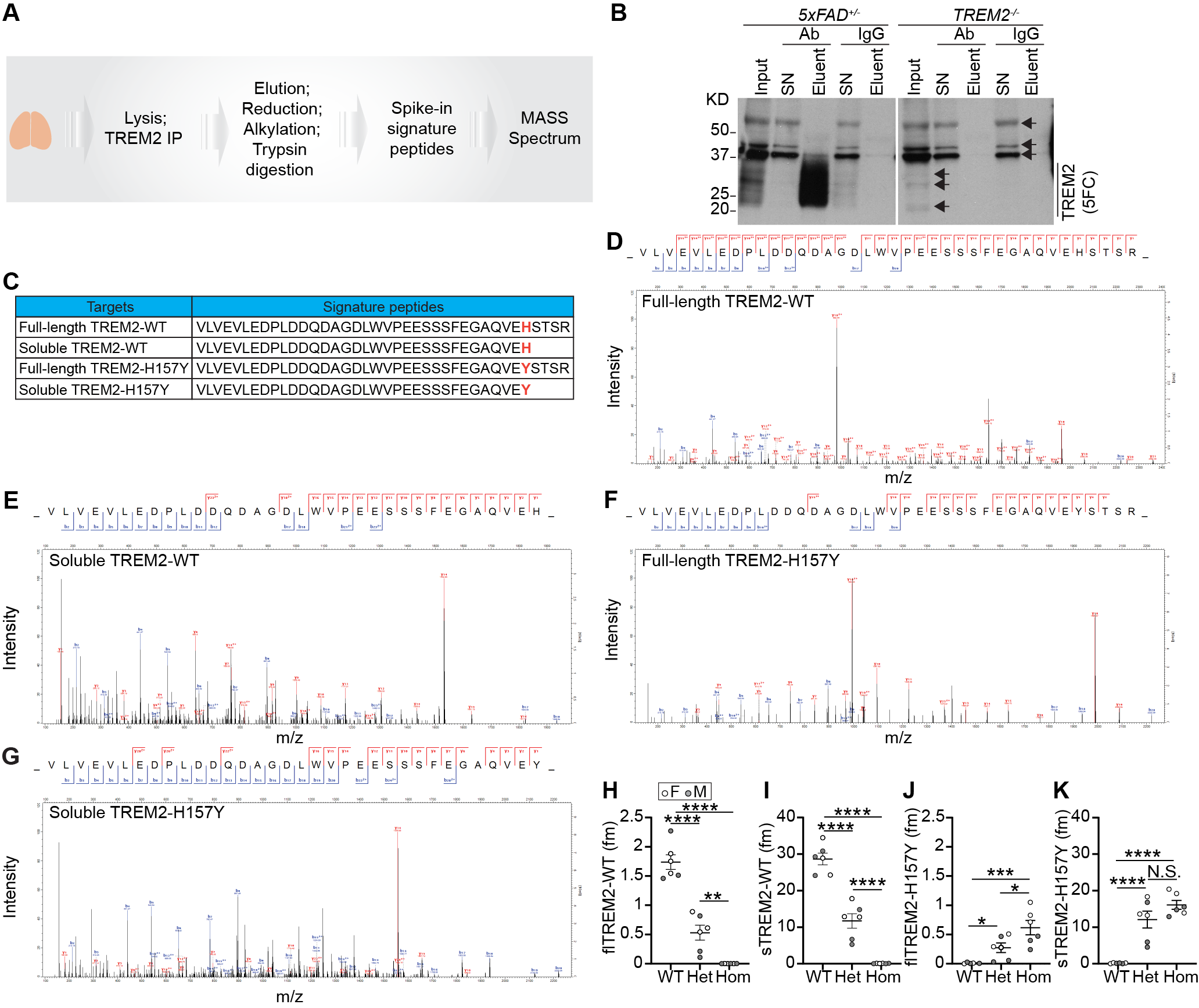

Supplement: Supplementary file 8 — Additional file 8: Figure S8. Identification and measurements of full-length, soluble TREM2-WT and TREM2-H157Y in mouse brain through mass spectrometry. A. Workflow of the TREM2-targeted mass spectrometry is illustrated. B. TREM2 was detected through an N-terminal antibody (5F4) in the input, supernatant and eluent samples from TREM2 immunoprecipitation through a biotinylated N-terminal antibody (BAF1729) in brain lysates of 5xFAD+/- and TREM2-/- mice. C. Signature peptide sequences for flTREM2-WT, sTREM2-WT, flTREM2-H157Y and sTREM2-H157Y are shown. D-G, Annotated MS/MS spectra of four unique peptides from flTREM2-WT (D), sTREM2-WT (E), flTREM2-H157Y (F) and sTREM2-H157Y (G), respectively. H-K. Measurements of flTREM2-WT (H), sTREM2-WT (I), flTREM2-H157Y (J) and sTREM2-H157Y (K) in brain samples from each genotype of 5xFAD mice. N = 3 mice/sex/genotype at 8.5 months of age. Data are presented as Mean±SEM. Ordinary one-way ANOVA with uncorrected Fisher's LSD multiple comparisons were used. N.S., not significant. *p < 0.01. **p < 0.01. ***p < 0.001. ****p < 0.0001. [file 13024_2023_599_MOESM8_ESM.png]

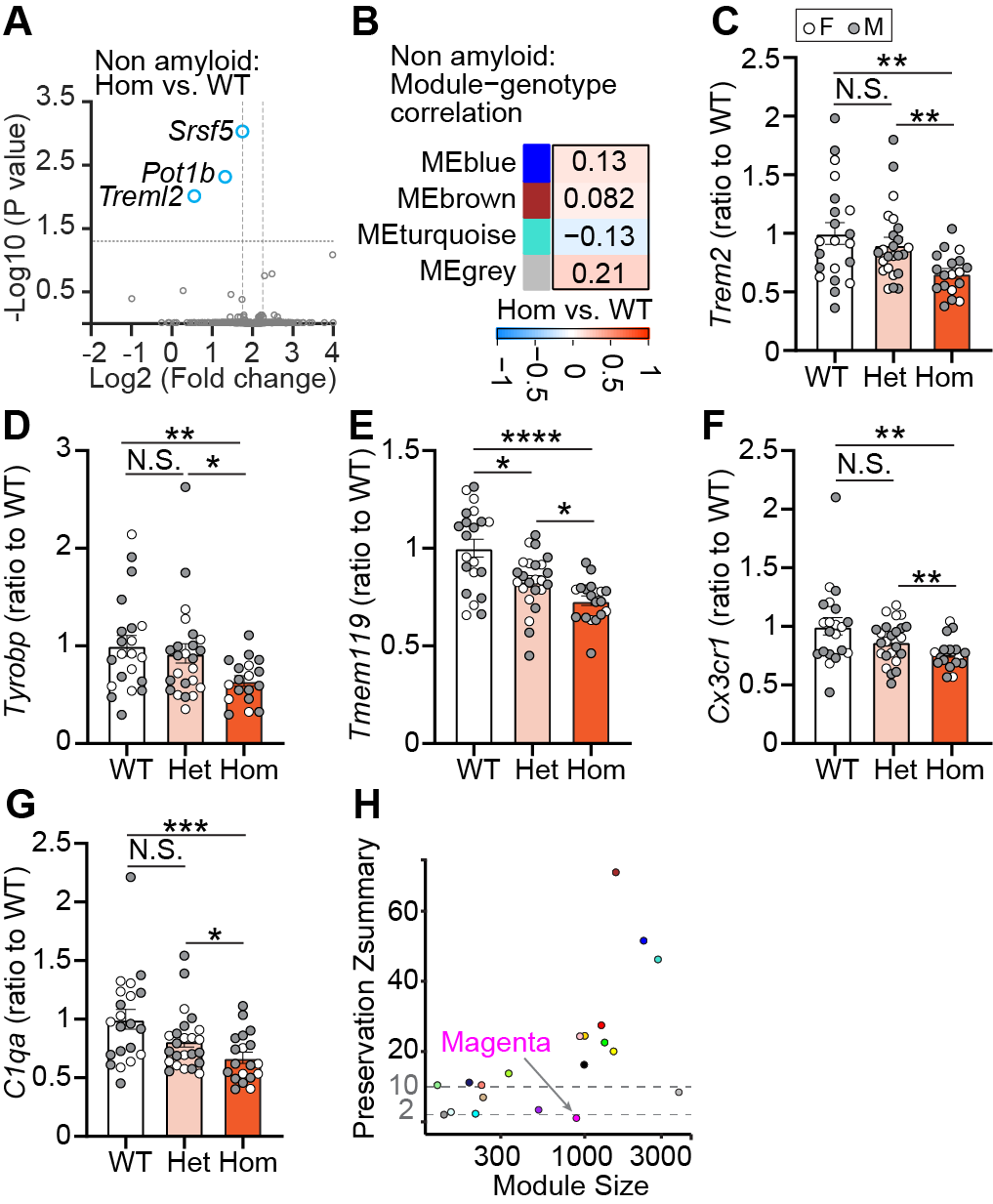

Supplement: Supplementary file 9 — Additional file 9: Figure S9. Effects of Trem2 H157Y on the transcriptome profiles. A. Three DEGs (Hom vs. WT, |Fold change|>1.2; FDR<0.05) were identified in the non-amyloid mice. N=5 mice/sex/genotype at 6 months of age. B. No significant modules were identified related to genotype in the non-amyloid cohort. C-G. DEGs, Trem2, Tyrobp, Tmem119, Cx3cr1, and C1q were validated through qPCR in 5xFAD mice. N = 19-24 mice per genotype at 8.5 months of age, mixed sex. Data are presented as Mean±SEM. Kruskal-Wallis tests with uncorrected Dun’s multiple comparisons were used. N.S., not significant. * p<0.05. ** p<0.01. *** p<0.001. **** p<0.0001. H. The magenta module identified in amyloid mice was not preserved in the non-amyloid network revealed by a low preservation Zsummary value (<2). [file 13024_2023_599_MOESM9_ESM.png]

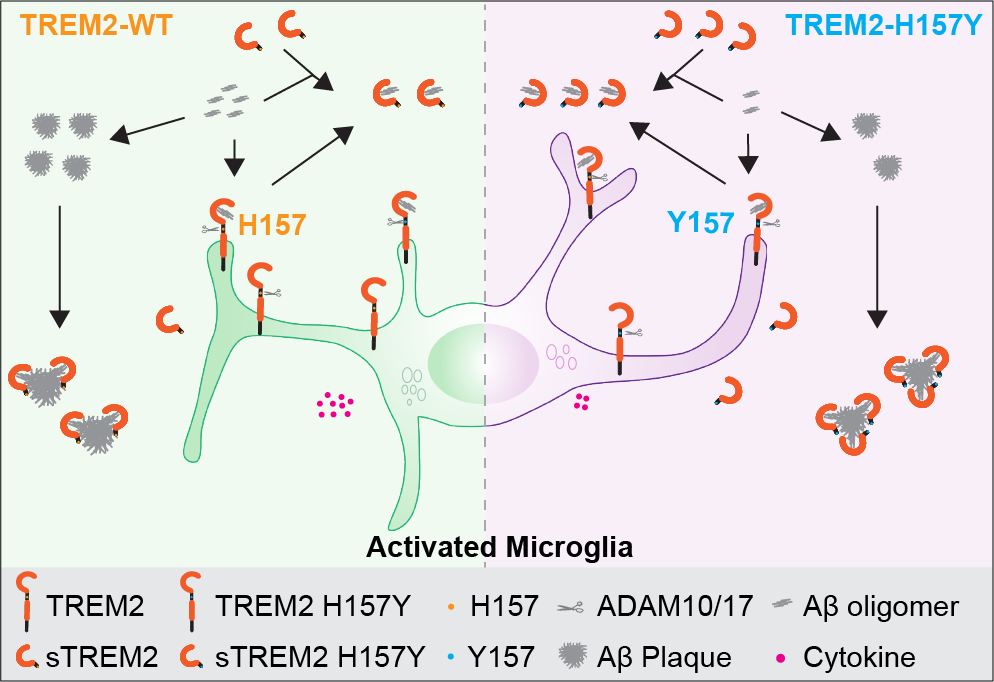

Supplement: Supplementary file 10 — Additional file 10: Figure S10. A working model is illustrated demonstrating the hypothesis that Trem2 H157Y reduces amyloid load through facilitating Aβ clearance mediated by sTREM2 which binds to Aβ oligomers and fibrils, and inhibit plaque formation. The reduced amyloid load leads to downregulated immune responses of microglia with less cytokine released. [file 13024_2023_599_MOESM10_ESM.png]
